# The Mating Competence of Geographically Diverse *Leishmania major* Strains in Their Natural and Unnatural Sand Fly Vectors

**DOI:** 10.1371/journal.pgen.1003672

**Published:** 2013-07-25

**Authors:** Ehud Inbar, Natalia S. Akopyants, Melanie Charmoy, Audrey Romano, Phillip Lawyer, Dia-Eldin A. Elnaiem, Florence Kauffmann, Mourad Barhoumi, Michael Grigg, Katherine Owens, Michael Fay, Deborah E. Dobson, Jahangheer Shaik, Stephen M. Beverley, David Sacks

**Affiliations:** 1 Laboratory of Parasitic Diseases, National Institute of Allergy and Infectious Diseases, National Institutes of Health, Bethesda, Maryland, United States of America; 2 Department of Molecular Microbiology, Washington University School of Medicine, St. Louis, Missouri, United States of America; 3 Biostatistics Research Branch, National Institute of Allergy and Infectious Diseases, National Institutes of Health, Bethesda, Maryland, United States of America; Duke University Medical Center, United States of America

## Abstract

Invertebrate stages of *Leishmania* are capable of genetic exchange during their extracellular growth and development in the sand fly vector. Here we explore two variables: the ability of diverse *L. major* strains from across its natural range to undergo mating in pairwise tests; and the timing of the appearance of hybrids and their developmental stage associations within both natural (*Phlebotomus duboscqi*) and unnatural (*Lutzomyia longipalpis*) sand fly vectors. Following co-infection of flies with parental lines bearing independent drug markers, doubly-drug resistant hybrid progeny were selected, from which 96 clonal lines were analyzed for DNA content and genotyped for parent alleles at 4–6 unlinked nuclear loci as well as the maxicircle DNA. As seen previously, the majority of hybrids showed ‘2n’ DNA contents, but with a significant number of ‘3n’ and one ‘4n’ offspring. In the natural vector, 97% of the nuclear loci showed both parental alleles; however, 3% (4/150) showed only one parental allele. In the unnatural vector, the frequency of uniparental inheritance rose to 10% (27/275). We attribute this to loss of heterozygosity after mating, most likely arising from aneuploidy which is both common and temporally variable in *Leishmania*. As seen previously, only uniparental inheritance of maxicircle kDNA was observed. Hybrids were recovered at similar efficiencies in all pairwise crosses tested, suggesting that *L. major* lacks detectable ‘mating types’ that limit free genetic exchange. In the natural vector, comparisons of the timing of hybrid formation with the presence of developmental stages suggest nectomonads as the most likely sexually competent stage, with hybrids emerging well before the first appearance of metacyclic promastigotes. These studies provide an important perspective on the prevalence of genetic exchange in natural populations of *L. major* and a guide for experimental studies to understand the biology of mating.

## Author Summary

*Leishmania* are pathogenic protozoa characterized by substantial diversity in the sand fly species that can transmit them, in the mammalian species that can serve as their reservoir hosts, and in the disease forms and severity of the clinical outcomes they can produce in humans. The possibility that this diversity has arisen, at least in part, by a process involving genetic exchange was recently given experimental support by the recovery of hybrid parasites from sand flies co-infected with two strains of *Leishmania major*. Here, we demonstrate the sexual competency of *L. major* strains originating across the full geographic range of this parasite species, and in both natural and unnatural sand fly vectors. Our genotype analyses of a large number of hybrid clones confirmed that they inherited both parental alleles at the majority of chromosomal marker loci analyzed, consistent with a meiotic process, while kinetoplast DNA was inherited from only one parent. Surprisingly, a few nuclear loci were sometimes inherited from only one parent, suggesting loss of heterozygosity. The early timing of hybrid recovery suggests that nectomonad promastigotes are the most likely mating competent stage of the parasite. These studies provide the strongest evidence to date that sex is a component of the natural reproductive strategy of *L. major*.

## Introduction

Protozoan parasites of the genus *Leishmania* present a remarkable epidemiologic and clinical diversity, producing a spectrum of human diseases that pose serious health concerns throughout tropical and sub-tropical regions. *Leishmania* have a dimorphic asexual life cycle consisting of extracellular promastigotes that multiply and develop within the alimentary tract of the sand fly vector, and intracellular amastigotes that multiply within the phagolysosomes of their host macrophages. No sexual dimorphism has been described, and based on the strong linkage disequilibrium revealed in population genetic studies applied to several *Leishmania* species, these parasites have been argued to be essentially clonal [1], [2]. This conclusion, however, must contend with a series of observations regarding the analysis of multilocus microsatellite, isoenzyme, or karyotype markers consistent with occasional sexual recombination and possible endogamy [3]–[8]. Many of these studies incorporated models that assumed *Leishmania* were ‘normal diploids’, which is now known to be untrue. Most and probably all *Leishmania* strains show varying degrees of aneuploidy (reviewed in [9], [10]). Moreover, chromosome number variation appears to be highly variable in culture, with individual cells/chromosomes showing monosomic, disomic and trisomic chromosome numbers [11]. The rapidity and transient nature of variable segregation, loss and expansion of chromosomes numbers are expected to have a profound effect on levels of heterozygosity, and to date the impact of rapidity and frequency of these factors on *Leishmania* has not been adequately accounted for by the population genetic models in the studies cited earlier. Nonetheless, it is clear from the literature that in natural populations hybrid parasites have been observed, and in some cases are widespread [12].

Two recent reports involving experimental crosses in sand flies have provided the first direct demonstration that genetic exchange in *Leishmania* can occur. The first series of successful crosses involved co-infection of *Phlebotomus duboscqi* sand flies using two strains of *L. major* engineered to express heterologous drug selectable markers [13]. The doubly drug-resistant clones obtained were clear genomic hybrids, based on the presence of both parental alleles at seven unlinked loci. Importantly, the same parental lines that successfully mated in the sand fly midgut failed to generate hybrid genotypes following their co-inoculation and high density growth in axenic culture or in the mouse ear dermis. From these data it was concluded that *L. major*, like *Trypanosoma brucei* [14], is capable of a sexual cycle consistent with a meiotic process, and that sex is confined to the extracellular stages of the parasite developing within the insect vector. A subsequent study by Sadlova *et al.* [15] used a fluorescent protein detection system to observe yellow promastigotes in *P. perniciosus* and *Lutzomyia longipalpis* midguts co-infected with RFP and GFP transgenic lines of *L. donovani*. The putative hybrids were observed in only two of the hundreds of co-infected flies examined, and the parasites could not be recovered and propagated to confirm their hybrid genotypes.

A number of essential features of genetic exchange in *Leishmania* remain to be further substantiated or explored, including the sexual competency of diverse *Leishmania* species and strains, the timing and frequency of hybrid formation in the midgut, their developmental stage associations, and the ability of other phlebotomine vectors to support genetic exchange. In the current studies, we have expanded the analysis of the mating competency of *L. major* strains to include pairwise matings of multiple isolates distributed over the full geographic range of this species. Our genotype analysis of a large number of progeny clones substantiates their chromosomal inheritance of both parental alleles at multiple unlinked loci, consistent with a meiotic process, and their uniparental inheritance of kinetoplast DNA. A low but unexpected frequency of nuclear loci were also inherited from only one parent, suggesting loss of heterozygosity. The timing of hybrid recovery in a natural vector suggests that nectomonad promastigotes are the most likely mating competent forms. Finally, we have demonstrated the capacity of a new world vector, *L. longipalpis*, to efficiently support the *Leishmania* sexual cycle.

## Results

### *L. major* crosses in *P. duboscqi*

Four different *L. major* strains derived from primary isolates originating in Senegal, Israel, Iraq, and southern Russia, and stably transfected with various antibiotic resistance markers, were tested for their ability to generate hybrid genotypes following co-infection of *P. duboscqi* sand flies, a natural vector of *L. major* transmission in Africa. In these experiments a HYG marker was integrated into the *LPG5A* locus, a SAT marker was integrated into one cistron within the rRNA locus, and the BSD marker was integrated into the *LPG5B* locus, as described previously [13], [16]. These loci were chosen as prior studies revealed no effects on parasite growth *in vitro* or in sand flies ([13] and data not shown). For all parental lines, a number of clonal lines were obtained and screened for their ability to show survival and complete development in *P. duboscqi*. Mating tests were performed by mixing equal numbers of parental lines, and feeding in vivo as described previously [13]. At varying times following the infective feed, midguts were dissected, parasites recovered, and cultured in media selective for the doubly-drug resistant hybrids. Flies cannot be maintained aseptically, and a variable proportion of the cultures established from the dissected midgut homogenates was lost to either fungal or bacterial contamination. The frequency of hybrid recovery was calculated as the percentage of the number of ‘clean’ midguts yielding doubly-drug resistant promastigotes. For each infected midgut yielding a doubly-drug resistant population, only a single clone was selected for further analysis. All lines studied are therefore a product of an independent mating event. In all selected clones, the presence of both parental drug markers was confirmed by PCR using primers specific for the respective antibiotic resistance markers (Table S1) (Fig S1). SNP typing of loci present on chromosomes unlinked to the integrated markers on the parental lines was performed subsequently.

Our initial crossing experiments employed the same *L. major* parental lines, Fn/Sat and Lv39c5/Hyg, used to generate the set of hybrids originally reported [13], in order to explore the timing of hybrid formation during *L. major* growth and development in *P. duboscqi*. Eight hybrids were recovered from the total of 112 clean midguts dissected on days 5, 9 and 13 post-infection (p.i.) (Table 1). Importantly, no hybrids were recovered from day 5 midguts, while 3 hybrids were recovered from day 9 (8.6%), and 5 from day 13 midguts (13.9%). Two additional timing studies were carried out using Fn/Sat paired with a new parental line, Sd/Hyg. In total, 15 hybrids were recovered amongst the 215 clean midguts dissected on days 4–15. Again, hybrids were recovered only at later time points, with no hybrids recovered on day 4 and only a single hybrid recovered on day 5 (2.4%). Flies dissected on days 6–8 yielded 7 hybrids (7.0%), while flies dissected on days 9–15 yielded 11 hybrids (10.3%). Thus, at least for mating pairs involving the Fn/Sat parental line for which the kinetics of hybrid formation in *P. duboscqi* was investigated, there was a significant correlation between the probability of hybrid recovery and the number of days of co-infection (Fn/Sat×Lv39/Hyg cross, p = 0.024; Fn/Sat×Sd/Hyg cross, p = 0.034; Fig S2).

**Table 1:** Timing of hybrid recovery in *P. duboscqi*.

Cross	Day post-infection	No. midguts dissected	No. clean midguts^1^	No. hybrids recovered	% hybrid recovery
Fn/Sat×Lv39c5/Hyg	5	48	41	0	0
	9	48	35	3	8.6
	13	48	36	5	13.9
Fn/Sat×Sd/Hyg	4	30	18	0	0
	8	39	25	1	4
	14	51	25	3	12
Fn/Sat×Sd/Hyg	5	48	41	1	2.4
	6	32	26	2	7.7
	8	48	38	3	7.9
	12	36	27	3	11.1
	15	15	15	2	13.3

1 Refers to the absence of fungal or bacterial contamination in the promastigote selection media containing the midgut homogenate.

As all of the successful mating attempts up to this point employed the Fn/Sat line as one of the parents, additional crosses were undertaken in *P. duboscqi* that did not involve the Fn strain. From two independent crosses involving LV39c5/Hyg and Sd/Bsd, we were able to recover 4 hybrids (6.7%) in flies dissected on days 8–10 p.i. A single experiment in flies co-infected with Lv39/Hyg and Ry/Sat yielded 5 hybrids (14.3%) from flies dissected day 10 p.i. A summary of all of the new crosses that we have undertaken in *P. duboscqi* involving the various pairwise combinations of the 4 *L. major* strains is presented in Table 2. For the sake of comparison, the summary table includes only flies that were co-infected for at least 8 days prior to dissection. Notably, the fraction of flies from which hybrids were recovered, ranging from 6.7–14.3%, did not vary significantly between crosses involving the different strains, including Fn vs both LV39c5 and Sd, or LV39c5 vs Sd, Ry and Fn. The findings suggest that *L. major* lacks detectable ‘mating types’ that might limit free genetic exchange.

**Table 2:** Summary of *L. major* crosses in *P. duboscqi*.

Cross	No. midguts dissected	No. clean midguts	No. hybrids recovered	% hybrid recovery
Fn/Sat×Lv39c5/Hyg	96	71	8	11.3
Fn/Sat×Sd/Hyg	248	157	15	9.5
Sd/Bsd×Lv39c5/Hyg	164	60	4	6.7
Ry/Sat×Lv39c5/Hyg	93	35	5	14.3

### Infection levels and development stage associations of hybrid formation

**Figure 1:**
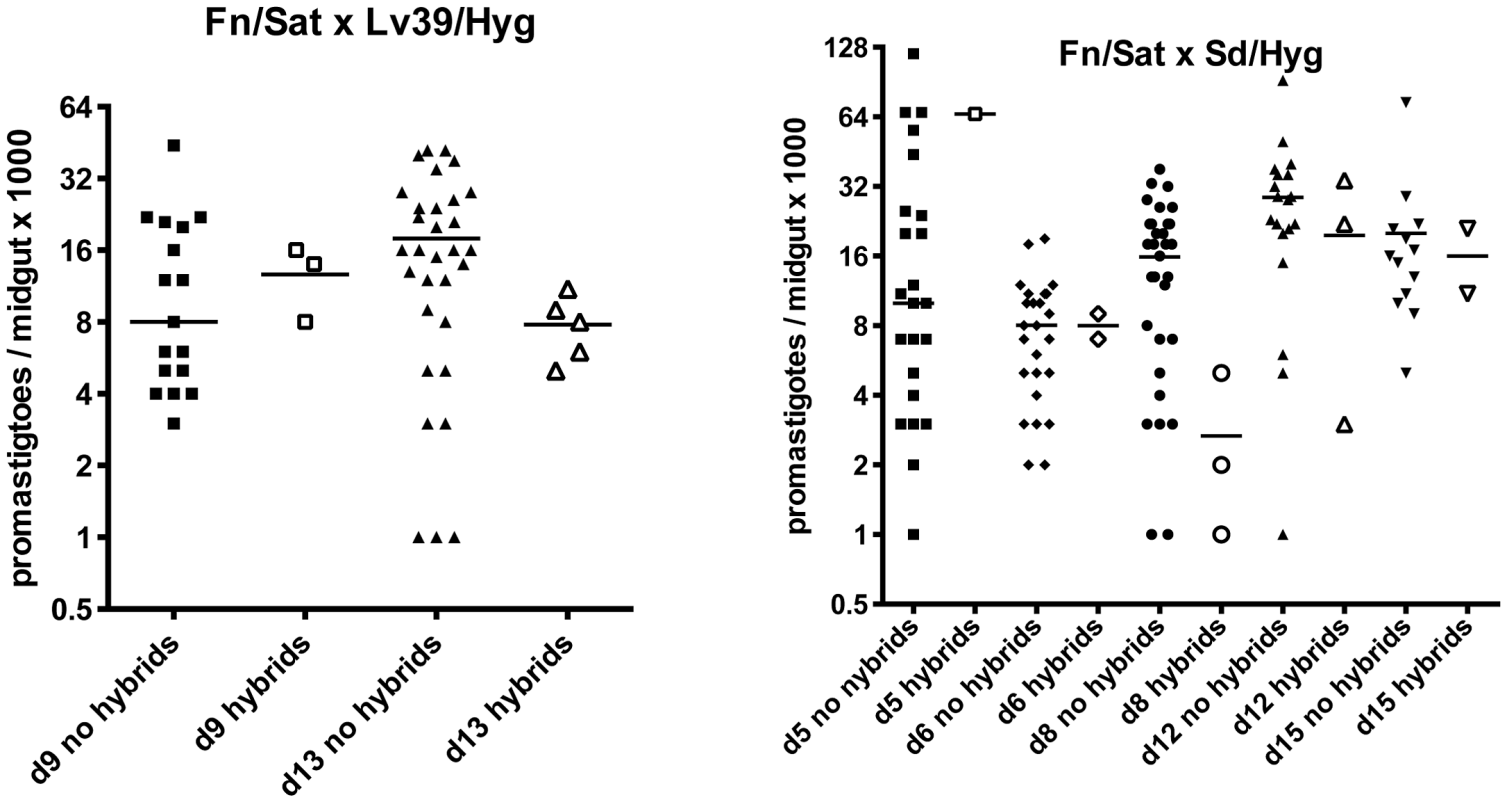
Infection levels at time of dissection in *P. duboscqi* midguts with and without hybrids. Midguts were dissected on the indicated times post-feeding and scored for the number of viable promastigotes. Bars represent the median numbers of promastigotes per midgut.

We addressed the question of whether there is a correlation between the intensity of infection in the midgut and the probability of hybrid recovery, by comparing the total number of promastigotes per gut at the time of dissection in flies yielding hybrids or not (Fig. 1). There was no significant difference between these groups. We were struck by the low infection levels even at late time points in many of the flies yielding hybrids (<10,000 promastigotes/gut).

In addition to infection levels, the association between hybrid formation and promastigote developmental stages was explored. During cyclical transmission in a competent vector, *Leishmania* promastigotes undergo a sequence of morphologic changes (reviewed in [17]). For *L. major*, these distinct forms typically include procyclics, which appear as short, ovoid, slightly motile and rapidly dividing cells that develop in the abdominal midgut during the first 72 hr. prior to their transformation to long, slender promastigotes, termed nectomonads, 3–6 days post-feeding. These forms fill the abdominal midgut, with many becoming attached by their flagellum to the microvillar lining. By 4–5 days, most of the digested bloodmeal is excreted and nectomonads can begin to be found in the thoracic midgut. This forward migration is accompanied by their gradual transformation over the following week to shorter, broader haptomonads attached to the intima of the stomodeal valve, as well as short, slender, highly active, free swimming and non-dividing metacyclic promastigotes that are the infective stage egested by the fly.

**Figure 2:**
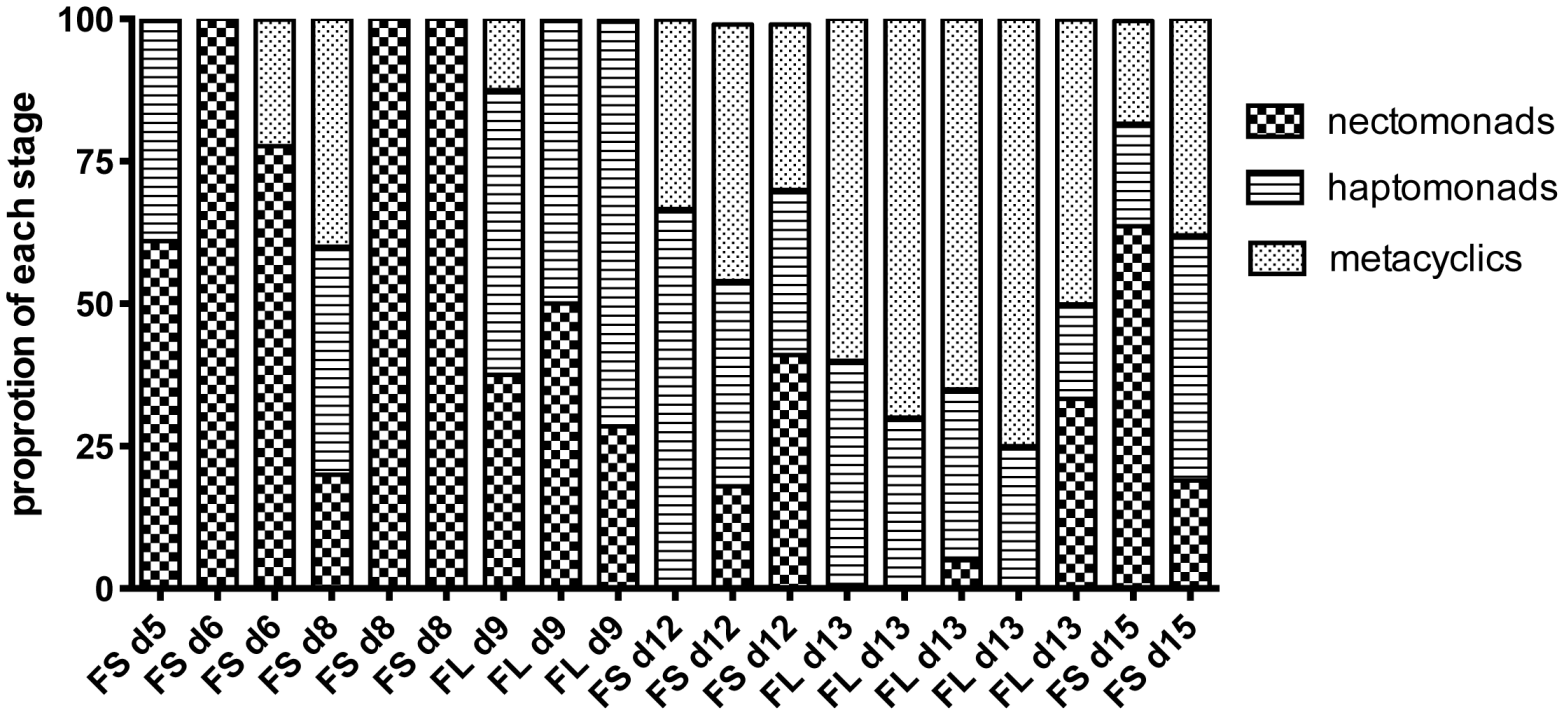
Proportion of promastigote developmental stages at the time of hybrid recovery in *P. duboscqi*. Midguts were dissected on the indicated times post-feeding and scored for the proportion of each developmental stage. Fn/Sat×Lv39c5 and Fn/Sat×Sd/Hyg hybrids are designated as FL and FS, respectively, followed by the day of hybrid recovery.

The proportion of these developmental forms at the time of dissection in each of the midguts from which hybrids were recovered from crosses involving Fn/Sat×Sd/Hyg or Fn/Sat×Lv39/Hyg is shown in Fig. 2. Even at the earliest time of hybrid recovery in a single fly on day 5, procyclics were no longer present and thus are not shown in the figure. This midgut, as well as 4 of the 5 midguts from which hybrids were recovered on days 6–8, was dominated by nectomonads, and 3 had exclusively these forms. The transition to infections dominated by haptomonad and metacylic promastigotes was observed in one midgut by day 8, but was otherwise delayed until day 9. By day 13, metacyclics were the predominant form in the midgut. Importantly, in 6 of the hybrid positive midguts dissected on days 5–9, no metacyclics were observed, indicating that metacyclogenesis is not a prerequisite for mating to occur. The increased frequency of hybrid recovery in flies with mature infections might reflect the greater opportunity that nectomonad-related forms have to mate, particularly as they continue to replicate and become more densely packed in the microenvironment of the anterior midgut. However, the contribution of late developmental stages, including metacyclics, to hybrid formation, cannot be discounted.

### Genotype analyses of hybrids generated in *P. duboscqi*

**Figure 3:**
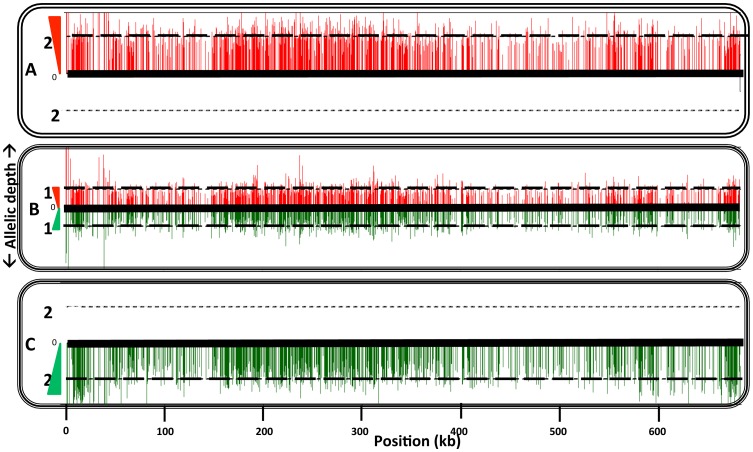
Comparison of homozygous parental SNPs on chromosome 17 of a representative hybrid. Homozygous parental SNP differences were identified following deep genomic DNA sequencing as described in the methods. SNPs mapping to the Fn line are shown in red and those mapping to Lv39c5 line are shown in green. The vertical distance corresponds to the inferred allelic depth, normalized across the entire genome, which was assigned an average ploidy of 2. The heavy dashed lines mark the value expected in the parents (2) or hybrid (1). Panel A, Fn; panel B, hybrid 5_22_A10 (recovered from *P. duboscqi*); panel C, LV39c5. Profiles of four additional representative hybrids can be found in Supplemental Figure S7. A total of 1314 homozygous SNP differences separate the parental Fn and LV39c5 lines.

As referred to above, PCR tests confirmed that in each case the progeny clones were true genetic hybrids bearing the parental selectable markers (Fig. S1). We also examined the segregation of loci not linked to the chromosomes bearing the drug resistance markers using SNPs identified previously [13] from comparisons of the *SCG* genes located on chromosomes 2, 7, 21, 25, 31, 35, and 36. Other nuclear markers were chosen based on SNPs previously identified by comparing different *L. donovani* isolates [18] and that we found to also identify allelic differences between some of the *L. major* sub-strains on chromosomes 14, 34. The remaining SNPs on chromosomes 4, 9, and 31 were identified by comparing gene alignments determined from the genome data bases of other *Leishmania* species, including *L. major*, *L. infantum*, *L. braziliensis*, and *L. mexicana*. The position and description of each SNP, and the primers and conditions used for their amplification, are summarized in Tables S1 and S2. The SNPs present in the different parents and their hybrid progeny were analyzed by direct sequencing (Fig S3), and are summarized for each of the crosses in Tables 3–6. Both parents were homozygous for every marker applied to the analysis of the hybrids generated by those particular parents. Of the 18 Fn/Sat×Sd/Hyg hybrids recovered, 15 were heterozygous at all 4 loci analyzed, while 2 were heterozygous at 3 loci, with the 4^th^ marker sequence not determined (Table 3). Interestingly, homozygosity was found for the FnSd1 hybrid for the marker on chromosome 35, and for the FnSd7d hybrid for the marker on chromosome 14. For the 8 hybrids generated from the Fn/Sat×Lv39c5/Hyg cross, heterozygosity was observed at all loci for which sequences were available, with at least 3 and in some cases all 5 of the loci showing both parental alleles (Table 4). For the 4 LV39c5/Hyg×Sd/Bsd hybrids, 3 were heterozygous at 5 of the loci analyzed, while the LSD2 hybrid was heterozygous at 4 loci, but was homozygous at the markers on chromosomes 9 and 31 (Table 5). Interestingly, the inheritance patterns at these two loci were from different parents. Finally, in the cross of Ry/Sat×LV39c5/Hyg, all 5 hybrids were heterozygous at all 5 loci analyzed (Table 6). In summary, taking into account both the drug-selectable and additional unlinked markers, all 35 progeny clones inherited both parental alleles present in at least 5 loci located on 5 different chromosomes, and 97% of the nuclear markers analyzed were heterozygous for these alleles. The progeny thus appear to be full genomic hybrids, with the caveat that the plasticity in chromosome number (or other process) might in some instances have resulted in loss of heterozygosity (LOH), reflected in the apparent uniparental inheritance at a few (<3%) of the marker loci analyzed. In our previous study LOH was not observed, most likely arising from the fact that fewer hybrids were studied. These conclusions are supported in preliminary genome-wide analysis of SNP inheritance, as deduced by whole genome deep sequencing (J. Shaik, N. Akopyants, D. Dobson, P. Lawyer, D. Elnaiem, D. Sacks, and S. Beverley, in preparation). Figure 3 and Figure S4 show an analysis of the inheritance of homogozygous parental SNP for *L. major* chromosome 17, showing biparental inheritance of 1314 SNPs for 5 representative hybrids.

**Table 3:** Summary of Fn/Sat×Sd/Hyg hybrid genotypes generated in *P. duboscqi*.

Line name	Day of hybrid recovery	HYG marker Chr 24	SAT marker Chr 27	Chr 14	Chr 34	Chr 35	Chr 36	Maxi circle	Ploidy
**Fn/Sat**	**-**	**HYG−**	**SAT+**	**Fn**	**Fn**	**Fn**	**Fn**	**Fn**	**2n**
**Sd/Hyg**	**-**	**HYG+**	**SAT−**	**Sd**	**Sd**	**Sd**	**Sd**	**Sd**	**2n**
FNSD1	6	HYG+	SAT+	H	H	**Fn**	H	Fn	2n
FNSD2	10	HYG+	SAT+	H	H	H	H	Sd	2n
FNSD3	10	HYG+	SAT+	H	H	H	H	ND	2n
FNSD4a	8	HYG+	SAT+	H	H	H	H	Fn	2n
FNSD4b	14	HYG+	SAT+	H	H	H	H	Fn	2n
FNSD4c	14	HYG+	SAT+	H	H	ND	H	Fn	2n
FNSD4d	14	HYG+	SAT+	H	H	ND	H	Fn	2n
FNSD5	10	HYG+	SAT+	H	H	H	H	Fn	**3n**
FNSD6a	8	HYG+	SAT+	H	H	H	H	Sd	2n
FNSD6b	8	HYG+	SAT+	H	H	H	H	Fn	2n
FNSD6c	8	HYG+	SAT+	H	H	H	H	Fn	**3n**
FNSD6d	12	HYG+	SAT+	H	H	H	H	Sd	2n
FNSD6e	12	HYG+	SAT+	H	H	H	H	Fn	2n
FNSD6f	12	HYG+	SAT+	H	H	H	H	Fn	2n
FNSD7a	5	HYG+	SAT+	H	H	H	H	Fn	**3n**
FNSD7d	6	HYG+	SAT+	**Sd**	H	H	H	Fn	**3n**
FNSD7e	15	HYG+	SAT+	H	H	H	H	Sd	**4n**
FNSD7f	15	HYG+	SAT+	H	H	H	H	Fn	**3n**

H, hybrid; ND, not determined.

**Table 4:** Summary of Fn/Sat×LV39c5/Hyg hybrid genotypes generated in *P. duboscqi*.

Line name	Day of hybrid recovery	HYG marker Chr 24	SAT marker Chr 27	Chr 4	Chr 10	Chr 31	Chr 34	Chr 35	Maxi circle	Ploidy
**Fn/Sat**	**-**	**HYG−**	**SAT+**	**Fn**	**Fn**	**Fn**	**Fn**	**Fn**	**Fn**	**2n**
**Lv39c5/Hyg**	**-**	**HYG+**	**SAT−**	**Lv**	**Lv**	**Lv**	**Lv**	**Lv**	**Lv**	**2n**
FL9D10	9	HYG+	SAT+	H	H	H	H	H	Fn	**3n**
Fl9E2	9	HYG+	SAT+	H	ND	H	H	H	Fn	2n
Fl9F6	9	HYG+	SAT+	H	ND	H	H	H	Fn	2n
FL13D6	13	HYG+	SAT+	H	H	H	H	H	Fn	2n
FL13D8	13	HYG+	SAT+	H	H	H	H	H	Fn	2n
FL13E3	13	HYG+	SAT+	H	ND	H	ND	H	Fn	2n
FL13E6	13	HYG+	SAT+	ND	H	H	H	H	Fn	2n
FL13F8	13	HYG+	SAT+	H	H	H	H	ND	Fn	2n

H, hybrid; ND, not determined.

**Table 5:** Summary of Sd/Bsd×LV39c5/Hyg hybrid genotypes generated in *P. duboscqi*.

Line name	Day of hybrid recovery	HYG marker Chr 24	BSD marker Chr 18	Chr 2	Chr 9	Chr 21	Chr 31	Chr 35	Chr 36	Maxi circle	Ploidy
**Sd/Bsd**	**-**	**HYG−**	**SAT+**	**Sd**	**Sd**	**Sd**	**Sd**	**Sd**	**Sd**	**Sd**	**2n**
**Lv39c5/Hyg**	**-**	**HYG+**	**BSD−**	**Lv**	**Lv**	**Lv**	**Lv**	**Lv**	**Lv**	**Lv**	**2n**
LSD2	10	HYG+	BSD+	H	**Sd**	H	**Lv**	H	H	Lv	2n
LSD4	8	HYG+	BSD+	ND	H	H	H	H	H	Sd	2n
LSD5	8	HYG+	BSD+	H	H	ND	H	H	H	Sd	2n
LSD6	8	HYG+	BSD+	H	H	ND	H	H	H	Sd	2n

H, hybrid; ND, not determined.

**Table 6:** Summary of Ry/Sat×LV39c5/Hyg hybrid genotypes generated in *P. duboscqi*.

Line name	Day of hybrid recovery	HYG marker Chr 24	SAT marker Chr 27	Chr 21	Chr 25	Chr 31	Chr 36	Chr 35	Maxi circle	Ploidy
**Ry/Sat**	**-**	**HYG−**	**SAT+**	**Ry**	**Ry**	**Ry**	**Ry**	**Ry**	**Ry**	**2n**
**Lv39c5/Hyg**	**-**	**HYG+**	**SAT−**	**Lv**	**Lv**	**Lv**	**Lv**	**Lv**	**Lv**	**2n**
RLV1	10	HYG+	SAT+	H	H	H	H	H	Ry	2n
RLV2	10	HYG+	SAT+	H	H	H	H	H	Ry	2n
RLV3	10	HYG+	SAT+	H	H	H	H	H	Ry	2n
RLV4	10	HYG+	SAT+	H	H	H	H	H	Ry	2n
RLV5	10	HYG+	SAT+	H	H	H	H	H	Ry	2n

H, hybrid.

The inheritance of the kinetoplast organelle in the hybrids was examined by analyzing the segregation of polymorphic regions within the maxicircle kDNA [9], [10] (Fig S5). In contrast to the inheritance of nuclear DNA, and consistent with our previous findings, maxicircle kDNA was inherited from a single parent (Tables 3–6). For the Fn/Sat×Sd/Hyg hybrids, 13 inherited both of the maxicircle kDNA markers analyzed from Fn, while 5 inherited the kDNA markers from Sd. The departure of this ratio from a random 50∶50 segregation is of borderline statistical significance (p = 0.096; exact two-sided binomial test). For the 8 hybrids generated in the Fn/Sat×Lv39c5/Hyg cross, all inherited both Fn kDNA markers. For the 5 Ry/Sat×Lv39c5/Hyg hybrids, all inherited both kDNA allelic markers from the Ry parent. Finally, only one kDNA marker was analyzed for the Lv39c5/Hyg×Sd/Bsd hybrids, with 3 inheriting the Sd marker and one the Lv39 marker.

DNA content analysis of the progeny clones revealed that while the majority resembled the ‘2n’ parents (recalling that while *Leishmania* chromosomes are predominantly diploid, aneuploidy is quite common), a number of polyploid progeny were observed, including a total of 6 ‘triploid’, and one ‘tetraploid’ hybrid (Fig. S6). DNA contents of intermediate amounts were not observed, although small differences cannot be ruled out. The ‘triploid’ clones remained ‘triploid’ following extensive serial *in vitro* passage and following recovery from mouse dermal lesions, whereas the single ‘tetraploid’ progeny clone remained ‘tetraploid’ *in vitro*, but only diploid cells that had lost their double drug resistance were recovered from infected mouse tissue.

We used SNP genotyping combined with cleaved amplified polymorphic site (SNP-CAPS) analysis performed as previously described [13], to determine the relative contribution of each parental genome in the triploid progeny. The analysis is based on a SNP in Glucose 6 phosphate dehydrogenase (G6PD-LmjF34.0080) in position 975 (Table S2) that results in a SACII restriction site in both Lv39/Hyg and Sd/Hyg and not in Fn/Sat. The bar graph in Fig S7 shows the ratio between the intensity of the uncut upper band from Fn/Sat and the middle band from either Lv39/Hyg or Sd/Hyg, and compares the various hybrids with controls generated by mixing the respective parental DNA at 1∶1, 2∶1 or 1∶2 ratios. The SNP-CAPS analysis confirms the direct sequencing results indicating that all of the progeny clones examined are allelic hybrids for this marker, and suggests that for the triploid progeny, the extra genome complement is inherited from Lv39/Hyg (Fig S7A) or from Sd/Hyg (Fig S7B), and in no instance from Fn/Sat, similar to the triploid hybrids previously described [13].

### *L. major* crosses in *Lu. longipalpis*

To confirm that mating in *Leishmania* can occur in another vector species, we pursued co-infections experiments in *Lu. longipalpis*, belonging to the genus *Lutozomyia*, and the chief vector of *L. infantum* transmission in the new world. The experiments were done using the two *L. major* strains Fn/Sat and Lv39c5/Hyg, which we know to be mating competent in *P. duboscqi* ([13]and this work). Although *Lu. longipalpis* is not a natural vector of *L. major*, it is permissive for the complete development of *L. major* [19], including the Fn/Sat and Lv39/Hyg lines (data not shown). An initial series of co-infection experiments were analyzed only at late time points (days 11–18) to optimize the chances of hybrid recovery (Table 7). In three experiments, a total of 10 doubly drug-resistant hybrids were recovered from 8–15% of the clean *Lu. longipalpis* midguts, similar to the percentage of hybrid recovery in *P. duboscqi* (Table 2). A kinetic experiment was undertaken to determine if the timing of hybrid formation might also be similar. Surprisingly, flies yielding hybrids were recovered in this experiment at a remarkably high frequency at all time points examined (40–65%), including already at day 3. A total of 51 doubly drug-resistant lines were established in this single kinetic study. Another striking feature of this experiment was the virtual absence of fungal or bacterial contaminants in the cultures established from the dissected midguts.

**Table 7:** Summary of Fn/Sat×Lv39c5/Hyg crosses in *Lu. Longipalpis*.

Expt #	Day post-infection	No. midguts dissected	No. clean midguts	No. hybrids recovered	% hybrid recovery
1	14	16	8	1	12
2	14	16	8	2	25
2	18	37	22	4	18
3	11	31	13	1	8
3	14	21	13	2	15
4	3	20	20	9	45
4	7	31	30	12	40
4	10	47	46	30	65

The 61 hybrids generated by the Fn/Sat×Lv39c5/Hyg crosses in *Lu. longipalpis* were cloned and genotyped using the markers previously described [13]. In addition to the drug resistance markers that were amplified by PCR, five different SNP markers on chromosomes 2, 7, 25, 35 and 36 and two SNP markers on maxicircle kDNA were analyzed, in this instance by SNP-CAPS analysis (Table 8). All of the hybrids carried both drug resistance markers, and the majority inherited both parental alleles at all 5 chromosomal markers, with heterozygous alleles present at 90% of the nuclear markers analyzed. Ten of the hybrids, however, were homozygous at 2, 3 or 4 of the 5 chromosomal marker loci analyzed. While for the kDNA markers uniparental inheritance was expected, it was surprising to find that all 61 of the hybrids had inherited their maxicircle kDNA from only the Fn parent. Finally, they all appeared to be ‘2n’, which again distinguishes the hybrids generated in these crosses from those arising in the natural vector *P. duboscqi* in this and especially the previous study [13].

**Table 8:** Summary of hybrid genotypes generated in *Lu. Longipalpis*.

Line name	Day of hybrid recovery	HYG marker Chr 24	SAT marker Chr 27	Chr 35	Chr 7	Chr 25	Chr 36	Chr 2	Maxi circle	ploidy
**Fn/Sat**	**-**	**HYG−**	**SAT+**	**Fn**	**Fn**	**Fn**	**Fn**	**Fn**	**Fn**	**2n**
**Lv39c5/Hyg**	**-**	**HYG+**	**SAT−**	**Lv**	**Lv**	**Lv**	**Lv**	**Lv**	**Lv**	**2n**
1.1	14	HYG+	SAT+	H	H	H	H	H	Fn	2n
2.2	14	HYG+	SAT+	H	H	H	H	H	Fn	2n
2.3	14	HYG+	SAT+	H	H	H	H	H	Fn	2n
2.4	18	HYG+	SAT+	H	H	H	H	H	Fn	2n
2.5	18	HYG+	SAT+	H	H	H	H	H	Fn	2n
2.6	18	HYG+	SAT+	H	H	H	H	H	Fn	2n
2.7	18	HYG+	SAT+	H	H	H	H	H	Fn	2n
3.8	11	HYG+	SAT+	H	H	H	H	H	Fn	2n
3.9	14	HYG+	SAT+	H	H	H	H	H	Fn	2n
3L3L6	3	HYG+	SAT+	H	H	H	H	H	Fn	2n
3L3A7	3	HYG+	SAT+	H	H	H	H	H	Fn	2n
3L3B4	3	HYG+	SAT+	**Fn**	H	H	**Fn**	H	Fn	2n
3L3B5	3	HYG+	SAT+	H	H	H	H	H	Fn	2n
3L3B11	3	HYG+	SAT+	H	H	H	H	H	Fn	2n
3L3C1	3	HYG+	SAT+	H	H	H	H	H	Fn	2n
3L3C6	3	HYG+	SAT+	H	H	H	H	H	Fn	2n
3L3C7	3	HYG+	SAT+	**Fn**	H	H	**Fn**	H	Fn	2n
3l3C8	3	HYG+	SAT+	H	H	H	H	H	Fn	2n
3L3D6	3	HYG+	SAT+	H	H	H	H	H	Fn	2n
3L3D7	3	HYG+	SAT+	H	H	H	H	H	Fn	2n
3L3E6	3	HYG+	SAT+	H	H	H	H	H	Fn	2n
3L7B4	7	HYG+	SAT+	H	H	H	H	H	Fn	2n
3L7B7	7	HYG+	SAT+	H	H	H	H	H	Fn	2n
3L7B9	7	HYG+	SAT+	H	H	H	H	H	Fn	2n
3LB11	7	HYG+	SAT+	H	H	H	H	H	Fn	2n
3L7C6	7	HYG+	SAT+	H	H	H	H	H	Fn	2n
3L7D8	7	HYG+	SAT+	H	H	H	H	H	Fn	2n
3L7D9	7	HYG+	SAT+	H	H	H	H	H	Fn	2n
3L7D10	7	HYG+	SAT+	H	H	H	H	H	Fn	2n
3L7D11	7	HYG+	SAT+	H	H	H	H	H	Fn	2n
3L7E6	7	HYG+	SAT+	H	H	H	H	H	Fn	2n
3L10A1	10	HYG+	SAT+	H	H	H	H	H	Fn	2n
3L10A2	10	HYG+	SAT+	H	H	**Fn**	H	**Fn**	Fn	2n
3L10A4	10	HYG+	SAT+	**Fn**	H	**Fn**	**Fn**	**Fn**	Fn	2n
3L10A5	10	HYG+	SAT+	H	H	H	H	H	Fn	2n
3L10A6	10	HYG+	SAT+	H	H	H	H	H	Fn	2n
3L10A8	10	HYG+	SAT+	H	H	H	H	H	Fn	2n
3L10A10	10	HYG+	SAT+	H	H	H	H	H	Fn	2n
3L10B3	10	HYG+	SAT+	H	H	H	H	H	Fn	2n
3L10B7	10	HYG+	SAT+	**Fn**	H	H	**Fn**	H	Fn	2n
3L10B10	10	HYG+	SAT+	H	H	H	H	H	Fn	2n
3L10B11	10	HYG+	SAT+	H	H	H	H	H	Fn	2n
3L10B12	10	HYG+	SAT+	H	H	H	H	H	Fn	2n
3L10C1	10	HYG+	SAT+	H	H	H	H	H	Fn	2n
3L10C4	10	HYG+	SAT+	H	H	H	H	H	Fn	2n
3L10C8	10	HYG+	SAT+	H	H	H	H	H	Fn	2n
3L10C11	10	HYG+	SAT+	H	H	H	H	H	Fn	2n
3L10D2	10	HYG+	SAT+	H	H	**Fn**	H	**Fn**	Fn	2n
3L10D3	10	HYG+	SAT+	H	H	H	H	H	Fn	2n
3L10D5	10	HYG+	SAT+	H	**Fn**	**Fn**	**Fn**	**Fn**	Fn	2n
3L10D7	10	HYG+	SAT+	H	H	**Fn**	**Fn**	**Fn**	Fn	2n
3L10D9	10	HYG+	SAT+	H	**Fn**	H	**Fn**	**Fn**	Fn	2n
3L10D12	10	HYG+	SAT+	**Fn**	**Fn**	H	**Fn**	H	Fn	2n
3L10E1	10	HYG+	SAT+	H	H	ND	H	H	Fn	2n
3L10E2	10	HYG+	SAT+	H	H	H	H	H	Fn	2n

H, hybrid; ND, not determined.

**Figure 4:**
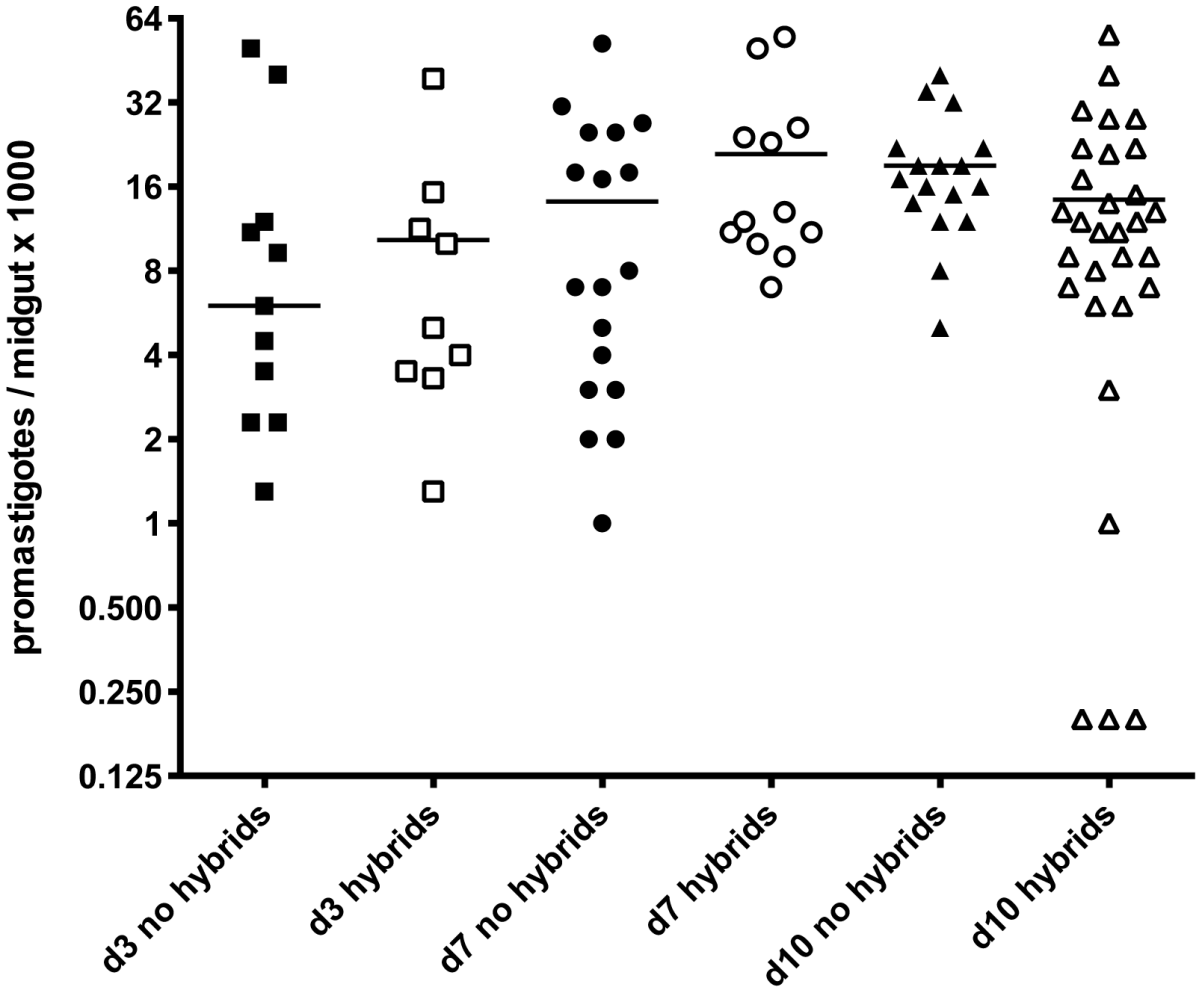
Infection levels at time of dissection in *Lu. longipalpis* midguts with and without hybrids. Midguts were dissected on the indicated times post-feeding and scored for the number of viable promastigotes. Bars represent the median numbers of promastigotes per midgut.

**Figure 5:**
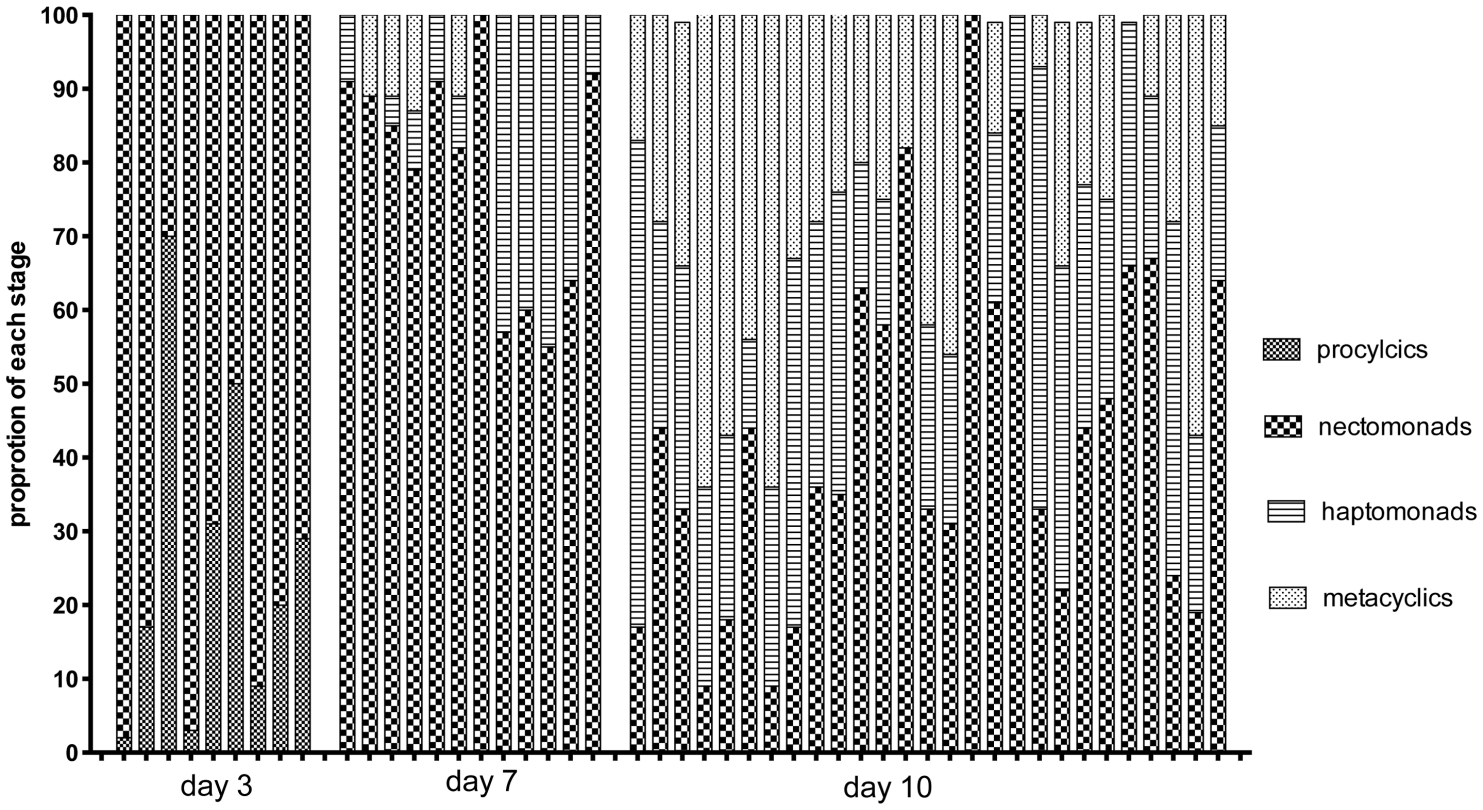
Proportion of promastigote developmental stages at the time of hybrid recovery in *Lu. Longipalpis*. Midguts were dissected on the indicated times post-feeding and scored for the proportion of each developmental stage.

The midguts analyzed in the kinetic study of hybrid formation in *Lu. longipalpis* were also used to determine the infection intensity and developmental stage associations in flies yielding hybrids or not. No significant difference in the number of promastigotes per midgut was observed between these groups, and hybrids were again recovered from many flies that had very low numbers of parasites, even at late time points (Fig. 4). There was also no difference at any time point between the hybrid positive and negative flies in the average number of each developmental stage (data not shown). Fig. 5 shows the relative proportion of each developmental stage at the time of dissection in the individual midguts yielding a hybrid. Because hybrids were recovered as early as day 3, procyclic forms were still present and may have been involved in hybrid formation. Multiple attempts in subsequent experiments to investigate hybrid recovery at days 1–2 when procyclics would have been the predominant if not exclusive stage in the gut, were negative. These experiments remain inconclusive, however, as the frequency of hybrid recovery even at late time points was very low (<5%), while the frequency of bacterial overgrowth in the midgut homogenate cultures was especially high (>80%) (data not shown). In the experiment shown in fig. 4, nectomonads were already the predominant stage in the majority of flies by day 3, consistent with prior observations regarding the accelerated progression of promastigote stage differentiation in *Lu. longipalpis* as compared to a natural vector [19], [20]. At day 7, nectomonads were still the prevailing form, and were found in all hybrid positive midguts at day 10, though in proportionately less numbers than haptomonads and metacyclics in most flies. Since the majority of the flies yielding hybrids at days 3 and 7 contained no metacyclics, the data again argue that metacyclogenesis is not required for mating competent forms to arise.

## Discussion

In this study we have extended and confirmed our original report regarding the capacity of *Leishmania* to undergo genetic exchange in the sand fly vector, analyzing in detail 96 independent hybrids. Using a system in which *P. duboscqi* sand flies, a natural vector of *L. major* transmission in West Africa, were co-infected with *L. major* strains expressing distinct drug selectable markers, we could confirm the mating capacity of strains originating throughout the geographic range of this species, from Senegal, West Africa to Israel, Iraq, and southern Russia. Hybrids were generated between all pairwise combinations tested, suggesting that barriers to mating between strains do not exist (or at least are not widespread). The proportion of flies yielding at least one hybrid was roughly comparable amongst the 4 mating pairs tested, and consistent with the estimates of a relatively low frequency of hybrid formation (<10^4^) described previously [13]. Mating must be considered as a non-obligatory part of the infectious cycle, since the majority of co-infected flies failed to yield a hybrid, yet were still permissive to the development of mature metacyclic forms. The results involving these geographically diverse *L. major* stocks, selected without bias, confirm that even if sexual reproduction is not obligatory and relatively rare, the machinery for genetic exchange has been maintained across the species. Additionally, we demonstrated the capacity of a new world phlebotomine vector, *Lu. longipalpis*, to support genetic exchange in *Leishmania*. Since genetic exchange has not been observed for promastigotes growing *in vitro*, the findings reinforce the conclusion that the phlebotomine midgut provides a unique environment for sex involving the insect stages of the parasite to occur.

For all of the hybrid progeny selected for double antibiotic resistance, the inheritance of both parental selectable markers was confirmed by PCR tests. Analysis of SNPs present on 4–6 additional chromosomes not linked to the selectable markers revealed that at 97% of the loci tested, progeny arising from crosses in the natural vector *P. duboscqi* inherited both parental alleles. However, 3% (4/150) loci showed loss of heterozygosity (LOH), inheriting only one allele from either parent (Tables 3–6). Curiously, taking into account both our current and prior studies involving the pairing of parental lines Fn/Sat and Lv39c5/Hyg in *P. duboscqi*, we found no examples of LOH (0/160 loci tested), whereas crosses of these same parents in the unnatural vector *Lu longipalpis* reported here, the frequency of LOH was 10% (27/275). These data suggest that the frequency of LOH may vary in individual crosses depending on both the parental lines and sand fly host. There are a number of mechanisms that could account for LOH – for example, meiotic or mitotic crossing over, or chromosomal segregation following mating associated with the rapidity by which *Leishmania* shows alterations in aneuploidy in culture [11], [21]. However, the overwhelming fraction of loci/hybrids show biparental inheritance. Correspondingly, , preliminary next gen sequencing data of the hybrids (J. Shaik, N. Akopyants, D. Dobson, P. Lawyer, D. Elnaiem, D. Sacks, and S. Beverley, in preparation), suggest that all hybrids recovered thus far arise biparentally across the entire genome (Fig. 3, S4). .

By contrast, maxicircle kDNA was again inherited in a uniparental manner, with a clear bias towards inheritance of kDNA from the Fn parent in crosses involving that parent. We have so far not been able to explore the possibility, documented for *T. brucei* [22], that recovery of hybrids during the earliest stage of their generation in the midgut might reveal biparental inheritance of kDNA, with subsequent loss during mitotic division.

Analysis of total DNA again revealed that while the majority of hybrids were ‘2n’, a number of ‘3n’ progeny were observed. We also recovered for the first time a single ‘4n’ hybrid (arising from a Fn/Sat×Sd/Hyg cross). Interestingly, while maintained stably as ‘4n’ in culture, this line reverted to ‘2n’ following recovery from an infected mouse. Tetraploid progeny had previously been seen in *Leishmania*, arising from attempts to inactivate essential genes [21]. In that study only one of 5 tetraploids tested remained infective to mice, however, following recovery this line remained tetraploid. Given the limited number of tetraploids obtained and examined thus far, further work will be required to systematically assess the stability and virulence of tetraploids in *Leishmania*.

In the studies here and previously reported [13], DNA contents of intermediate contents were not observed, although changes involving aneuploidy of a small number of chromosomes would not have been revealed by the methods used. These data could imply that meiosis occurs prior to or after a fusion event involving ‘1n’ cells or nuclei, with ’3n’ progeny resulting from the absence or incomplete meiotic division of one of the diploid parents. The ‘4n’ hybrid is most readily explained by fusion of diploid cells, and may represent an intermediate sexual stage that undergoes reduction to the diploid state, similar to the process described for *Candida albicans* [23]. Polyploid hybrids have been recovered in African trypanosomes, and inferred for American trypanosomes assuming *T. cruzi* undergoes reduction to an aneuploid state via a parasexual process [24]. For *T. brucei*, Mendelian inheritance patterns are well-established [25] and an epimastigote life-cycle stage residing in the tsetse fly salivary glands has recently been identified expressing meiotic genes [26]. Importantly, the expression of meiosis-specific proteins in most cases occurred prior to cell fusion, establishing a precedent in excavate protists for conventional meiotic division, although it should be noted that haploid trypanosomes have still not been formally demonstrated in *T. brucei*. The meiosis specific orthologs that were used to detect meiotic forms in *T. brucei* are also present in the *L. major* genome [27], and we are currently investigating their expression during development of midgut forms *in vivo*.

The second major finding arising from our studies pertains to the timing and developmental stage associations of hybrid formation in *P. duboscqi* sand flies. In 3 independent mating experiments investigating the timing of hybrid generation, only 1 hybrid was recovered amongst the 100 co-infected midguts dissected prior to day 6 (1%). Ten hybrids were recovered amongst the 134 guts dissected on days 6–9 (7.5%), and 15 of 112 (13.4%) on days 10–15. The delay in hybrid recovery strongly suggests that the rapidly dividing procyclic forms predominating in the midgut during days 1–2 post-infection are unlikely to be mating competent, especially as these forms have completely transitioned into nectomonads by days 4–5. The increased frequency of hybrid recovery on days 6–9 coincides with the colonization of the posterior midgut by primarily nectomonad forms and their initial migration to the thoracic midgut. The highest rate of hybrid recovery observed at later time points (days 10–15) might implicate more mature stages, e.g. haptomonads or metacyclics, as mating competent forms, or simply reflect the greater opportunity that nectomonads that are maintained in variable numbers even at late time points might have to mate, especially as they become more densely packed in the anterior gut. Since the flies from which hybrids were recovered days 5–9 post-infection harbored few and in most cases no metacyclics, it can be concluded that metacyclogenesis is not a pre-requisite for mating to occur. The intensity of infections at the time of dissection was also not a good predictor of mating success, as the total number of promastigotes per midgut was no higher in flies yielding hybrids compared to those that did not, and in some cases hybrids were recovered from flies that had very light infections (<2000 promastigotes/midgut). In summary, neither the earliest dividing procyclic forms, nor the late developing, resting metacyclic forms, appear to be associated, or uniquely so, with hybrid formation, consistent with the evidence against mating involving these analogous life cycle stages in *T. brucei* [26], [28], [29]. We favor a role for nectomonad-related forms in mating based not only on the timing of hybrid recovery, but also the fact that these forms are unique to *L. major* stage differentiation *in vivo* and are not observed amongst the pleopmorphic promastigotes that appear during growth in culture, which have so far remained mating incompetent. Nectomonads also share with *T. brucei* epimastigotes the ability of their flagellum to mediate attachment to the substratum, which involves adhesion to the midgut epithelium for *Leishmania*, and to the salivary gland epithelium for African trypanosomes. By analogy with the process of gamete activation and fusion in other flagellates, e.g. *Chlamydomonas* [30], such junctional complexes may inititate the signaling events that are required for hybrid formation in *Leishmania* and African trypanosomes to occur [29].

Despite the fact that *Lu. longipalpis*, which is a natural vector of *L. infantum* in the new world, does not naturally transmit *L. major*, it is permissive to the full development of this species in the laboratory [19]. The current studies confirm that it also supports genetic exchange in *L. major*. Genotype analysis of 61 hybrids revealed that in each case they were full genomic hybrids, since they possessed both parental alleles at unlinked loci on 7 different chromosomes, but again with the caveat that heterozygosity appears to have been lost at a number of loci. The inheritance bias of Fn maxicircle kDNA observed in hybrids generated in *P. duboscqi* was for the *L. longipalpis* hybrids complete, with all 61 progeny clones inheriting maxicircle kDNA exclusively from the Fn parent. The mechanisms that might account for the elimination of one of the kinetoplast organelles pre- or post-fusion, and how this process can be so selective, are currently unknown.

In an initial series of experiments involving flies dissected only at late time points, the efficiency of hybrid recovery was roughly comparable to that observed in *P. duboscqi*, with an average of 15% of flies with mature infections yielding at least one hybrid progeny. When a time course study was carried out in *Lu. longipalpis* in order to determine when hybrid formation might begin to occur in these flies, we were surprised to recover hybrids from a high proportion of flies (45%) already by day 3. This remarkable rate of hybrid recovery was maintained or increased at the later time points. In subsequent experiments involving different populations of released adults from the same colony, we failed to observe such a high rate of hybrid recovery (data not shown). While we cannot explain what remains so far a unique experience involving these flies, we note that the released adults used in the successful time course experiment were unusual in the virtual absence of any midguts harboring commensal bacteria that overgrew the promastigote growth medium containing penicillin and streptomycin. The possible influence of the midgut microbiota on promoting or inhibiting hybrid formation is currently being investigated.

Regarding the early appearance of hybrids by day 3, we and others [19], [20] have observed that the sequence of promastigote stage differentiation proceeds more rapidly in *Lu. longipalpis* compared with *P. duboscqi* or *P. papatasi*, with nectomonads the dominant stage already by day 3, and fully mature metacyclics the predominant stage already by day 8. Nonetheless, we cannot rule out the contribution of the earliest procylic promastigote stage to hybrid formation in *Lu. longipalpis*, which would be consistent with the timing and morphology of the putative *L. donovani* hybrid observed in the single *Lu. longipalpis* fly reported by Sadlova et al. [15].

In summary, our analysis of a large number of hybrid progeny generated between multiple strains of *L. major* substantiates that sex is a normal aspect of their reproductive strategy. The timing of hybrid recovery in a natural vector implicates the involvement of nectomonad forms in hybrid formation. Our finding that *Leishmania* crosses can be achieved experimentally in a proven vector of the genus *Luztomyia*, is highly relevant to a number of population studies showing the widespread occurrence of hybrid genotypes in New World *Leishmania* [3]–[8].

## Materials and Methods

### Ethics statement

This study was carried out in strict accordance with the recommendations in the Guide for the Care and Use of Laboratory Animals of the National Institutes of Health. The protocol was approved by the Animal Care and Use Committee of the NIAID, NIH (protocol number LPD 68E). Invertebrates are not covered under NIH guidelines.

### Parasites

*L. major* Fn/Sat was derived from a strain (MHOM/IL/80/Friedlin) originally isolated from a patient with cutaneous Leishmaniasis acquired in the Jordan Valley, and contains a heterozygous nourseothricin–resistance (SAT) marker, integrated along with a linked firefly luciferase reporter gene into one allele of the ∼20 rRNA cistrons located on chromosome 27 [31] using constructs and methods described previously [13]; *L. major* LV39c5/Hyg was derived from a strain originally isolated from a reservoir rodent host in southern Russia (MHOM/Sv/59/P) [32] and is heterozygous for an allelic replacement of the *LPG5A* on chromosome 24 by a hygromycin B–resistance cassette [16]; *L. major* Sd/Hyg was derived from a strain isolated from a patient with cutaneous lesions acquired in Senegal (MHOM/SN/74/SD) [33], and is also heterozygous for an allelic replacement of the *LPG5A* on chromosome 24 by a hygromycin B–resistance cassette [16]; *L. major* Sd/Bsd is derived from the same Sd strain from Senegal and is heterozygous for allelic replacement of the *LPG5B* on chromosome 18 by a blastocidin-resistance cassette [16]; *L. major* Ry/Sat was derived from a strain originally isolated from a lesion biopsy of a laboratory worker accidentally exposed to sand flies that were experimentally infected with a strain of *L. major* (WR2885) originating in Iraq [34], and also contains a heterozygous SAT–resistance marker, integrated into one allele of the rRNA cistrons located on chromosome 27 using constructs and methods described previously [13]. All parasites were grown at 26°C in complete medium 199 (CM199) supplemented with 20% heat-inactivated FCS, 100 U/ml penicillin, 100 µg/ml streptomycin, 2 mM L-glutamine, 40 mM Hepes, 0.1 mM adenine (in 50 mM Hepes), 5 mg/ml hemin (in 50% triethanolamine), and 1 mg/ml 6-biotin, and containing either 25 ug/ml blasticidin S (Invitrogen, Carlsbad, CA), 25 ug/ml hygromycin B (EMD Biosciences, San Diego, CA) or 100 ug/ml SAT (Jena Bioscience, Germany), or combinations of these antibiotics as necessary.

### Sand fly infections and hybrid recovery

Three to five day old female *P. duboscqi* or *L. longipalpis* sand flies, obtained from colonies initiated from field specimens collected in Mali and Brazil, respectively, were fed through a chick skin membrane on heparinized mouse blood containing 1–4×10^6^/ml logarithmic phase promastigotes of each parental line of *L. major*. At various days after the infective feed (3–18 days), the sand flies were anesthetized with CO_2_, their midguts dissected and homogenized in 100 µl CM199, and the number of viable promastigotes and the percentage of each promastigote developmental stage were determined by counting under a hemacytometer. Promastigote stages were identified based on the morphologic criteria previously described [35]. For isolation of hybrids, individual midgut homogenates from co-infected flies were transferred to a single well of a 96 well flat bottom plate, and incubated at 26°C over night. An equal volume of CM199 containing 2× of the appropriate antibiotics was added on the following day for the selection of hybrids. Doubly drug-resistant promastigotes were cloned by distribution in 96 well blood agar plates in 0.1 ml CM199 containing both antibiotics. Poisson analysis was used to determine the percentage probability of clonality, and was in each case >95%.

### DNA extraction, PCR and genotyping of hybrids

Parasite DNA was extracted as follows: cell extracts were suspended in TELT (0.05 M Tris, 005 M EDTA, 0.25 M LiCl and Triton X-4) and supplemented with an equal Volume of Phenol Chloroform isoamyl alcohol (25∶24∶1). Cell suspensions were centrifuged (15′ 16,000 g 4°C) and the aqueous phase was transferred to a new tube. DNA was precipitated in 96% ethanol and washed with 70% ethanol. DNA was then eluted with 100 ul of nuclease free water. For hybrids genotyping, a set of marker genes (Table S1) was amplified by polymerase chain reaction (PCR) using Applied Biosystems 2× geneAmpPCR mix, 25 pmol of each primer and 10 ng of the DNA. PCR products were cleaned with ExoSAP-IT kit (USB), and sequences confirmed with forward and/or reverse reads by Rocky Mountain Laboratory Genomics Unit DNA Sequencing Center, Division of Intramural Research, Hamilton, Montana. The sequences were analyzed using “Lasergene” software. SNP-CAPS analysis was performed as previously described [9] and was based on SNPs identified in the sequence analysis. Glucose phosphate dehydrogenase (G6PD-LmjF34.0080) was PCR-amplified and cleaned using gel and PCR clean up system (Promega). The clean PCR product (500 ng) was digested with 15 units of SACII (Thermo scientific) for 16 hours. Cleaved DNA was then loaded on ethidium bromide gel. Band intensity quantification was done using image J software (http://rsbweb.nih.gov/ij, National Institutes of Health, Bethesda, MD, USA).

### DNA content

DNA content was determined using flow cytometry as previously described [21]. Briefly, 2.5×10^6^ log-phase promastigotes were permeabilized with 4% paraformaldehide solution for 1 minute. Cells were centrifuged, re-suspended in PBS and fixed with absolute methanol for 15 minutes on ice. Cells were washed and resuspended in PBS at room temperature for 10 minutes followed by RNAseA treatment (200 µg/ml RNase A) and propidium iodide (20 µg/ml) staining for 30 minutes at room temperature. Data were acquired on a FacsCANTO 2 flow cytometer (BD Bioscience), counting at least 10,000 cells per sample, and analyzed using FlowJo 9.5.2 software.

### SNP analysis

The full methods and analysis of the parental and hybrid *L. major* lines studied here will be presented elsewhere (J. Shaik, N. Akopyants, D. Dobson, P. Lawyer, D. Elnaiem, D. Sacks, and S. Beverley, in preparation). Briefly, Leishmania nuclear DNA was isolated and subjected to deep-sequencing using Illumina Hi-seq 2000 machine, yielding approximately 60× coverage. Reads were aligned to the *L. major* reference genome (REF) using novoalign MPI version 2.07.07 (Novocraft, 2012); “NovoAlign (http://www.novocraft.com).”) and SNPs were identified using the Samtools pileup function using SOAP SNP consensus model [36]. Custom scripts were developed for subsequent analysis and display. SNPs were filtered to remove those falling within regions of low coverage (read depth less than 10) and to remove SNPs heterozygous within either parent. For the remaining homozygous SNPs, an ‘allelic’ density at each homozygous SNP position was estimated, assuming an average ploidy of 2n across the *L. major* genome. While not strictly true for any given parent or hybrid line (all of which vary from each other), the error introduced by this assumption is small. The results of these analyses are displayed as ‘bottlebrush’ plots (Fig. 3, Fig. S4) where at each SNP position the inferred allelic density is displayed on the Y-axis.

### Statistics

To test if there is a change over time in the proportion of hybrids recovered within each cross, we used an exact two-sided Cochran-Armitage test for trend using the day as the score combining across experiments and assuming no experiment effects. To compare the number of promastigotes per gut between flies yielding hybrids versus those not yielding hybrids, we used a stratified (by day) Wilcoxon-Mann-Whitney test. Tests were performed using SAS Version 9.3 (Cochran-Armitage) or the coin package in Hothorn et al. [37]. URL http://www.jstatsoft.org/v28/i08/.].

## Supporting Information

Figure S1 PCR for parental selectable drug markers. Samples are (A) Fn, Fn/Sat; Sd, Sd/Hyg; -, no template control; (B) Ry, Ry/Sat; Lv, Lv39/Hyg; -, no template control; (C) Lv, Lv39/Hyg; Sd, Sd/BSD; -, no template control; (D) Fn, Fn/Sat; Lv, Lv39/Hyg; -, no template control.
(PDF)

Figure S2 Plot of nonparametric maximum likelihood estimators of the proportion of flies with any hybrid by day for each of the crosses (solid lines), along with 95% pointwise confidence intervals (dotted lines). The confidence intervals use nonparametric bootstrap with a necessary adjustment for the upper interval for early days using an exact binomial upper interval.
(PDF)

Figure S3 DNA sequence traces of PCR-amplified chromosomal genes showing inheritance of both parental alleles. DNA sequence traces are shown for 4 representative loci and 16 representative 2n hybrids and their respective parents. SNPs are identified by the boxed regions.
(PDF)

Figure S4 Comparison of homozygous parental SNPs on chromosome 17 for four additional representative hybrids. Please refer to the legend for Figure 3 for a description of the methods and symbols used. Panel A and B, hybrids recovered from *P. duboscqi* (1_10_B12 and 4_7_A3). Panel C and D, hybrids recovered from *L. longipalpais* (LL1_3 and 3L3c1).
(PDF)

Figure S5 DNA sequence traces of PCR-amplified maxicircle genes showing inheritance of a single parental allele. DNA sequence traces are shown for representative loci in the maxicircle genes (1 SNP in the 12S rRNA locus, and 2 SNPs in the ND-5 locus) and 16 representative 2n hybrids and their respective parents. SNPs are shown in boxed regions.
(PDF)

Figure S6 DNA contents of parental Fn/Sat and Sd/Hyg (A) , and representative ‘2n’ (B), ‘3n’ (C), and ‘4n’ (D) hybrid clones. DNA content was measured in log phase cells by flow cytometry after staining RNase treated permeabilized cells with propidium iodide.
(PDF)

Figure S7 SNP-CAPS analysis of triploid hybrids. (A,B) Digestion with SACII of the G6PD-LmjF34.0080 divergent region PCR product is shown. (C,D) The graph shows the ratio between the intensity of the uncut upper band from Fn (F) and the middle band from either Lv39 (L) or Sd (S), and compares the various hybrids with controls.
(PDF)

Table S1 The PCR primer sequences for each allelic marker, their chromosomal location, and the conditions used for their amplification are shown. Δ - Genebank: M10126.1; nd, not determined.
(PDF)

Table S2 The chromosomal location, position and description of each SNP are shown. Position of SNPs in the maxicircle genes refer to *L .tarentolae* kDNA. Genbank: M10126.1.
(PDF)

## References

[1] Tibayrenc M, Ben Abderrazak S, Guerrini F, Banuls A (1993) Leishmania and the clonal theory of parasitic protozoa. Arch Inst Pasteur Tunis 70: 375–382. 7802492

[2] Tibayrenc M, Kjellberg F, Ayala FJ (1990) A clonal theory of parasitic protozoa: the population structures of Entamoeba, Giardia, Leishmania, Naegleria, Plasmodium, Trichomonas, and Trypanosoma and their medical and taxonomical consequences. Proc Natl Acad Sci U S A 87: 2414–2418. 2320563 10.1073/pnas.87.7.2414 PMC53699

[3] Banuls AL, Guerrini F, Le Pont F, Barrera C, Espinel I, et al (1997) Evidence for hybridization by multilocus enzyme electrophoresis and random amplified polymorphic DNA between Leishmania braziliensis and Leishmania panamensis/guyanensis in Ecuador. J Eukaryot Microbiol 44: 408–411. 9304809 10.1111/j.1550-7408.1997.tb05716.x

[4] Bastien P, Blaineau C, Pages M (1992) Leishmania: sex, lies and karyotype. Parasitol Today 8: 174–177. 15463609 10.1016/0169-4758(92)90016-u

[5] Nolder D, Roncal N, Davies CR, Llanos-Cuentas A, Miles MA (2007) Multiple hybrid genotypes of Leishmania (viannia) in a focus of mucocutaneous Leishmaniasis. Am J Trop Med Hyg 76: 573–578. 17360886

[6] Ravel C, Cortes S, Pratlong F, Morio F, Dedet JP, et al (2006) First report of genetic hybrids between two very divergent Leishmania species: Leishmania infantum and Leishmania major. Int J Parasitol 36: 1383–1388. 16930606 10.1016/j.ijpara.2006.06.019

[7] Rougeron V, Banuls AL, Carme B, Simon S, Couppie P, et al (2011) Reproductive strategies and population structure in Leishmania: substantial amount of sex in Leishmania Viannia guyanensis. Mol Ecol 20: 3116–3127. 21722225 10.1111/j.1365-294X.2011.05162.x

[8] Rougeron V, De Meeus T, Hide M, Waleckx E, Bermudez H, et al (2009) Extreme inbreeding in Leishmania braziliensis. Proc Natl Acad Sci U S A 106: 10224–10229. 19497885 10.1073/pnas.0904420106 PMC2700931

[9] Mannaert A, Downing T, Imamura H, Dujardin JC (2012) Adaptive mechanisms in pathogens: universal aneuploidy in Leishmania. Trends Parasitol 28: 370–376. 22789456 10.1016/j.pt.2012.06.003

[10] Sterkers Y, Lachaud L, Bourgeois N, Crobu L, Bastien P, et al (2012) Novel insights into genome plasticity in Eukaryotes: mosaic aneuploidy in Leishmania. Mol Microbiol 86: 15–23. 22857263 10.1111/j.1365-2958.2012.08185.x

[11] Sterkers Y, Lachaud L, Crobu L, Bastien P, Pages M (2011) FISH analysis reveals aneuploidy and continual generation of chromosomal mosaicism in Leishmania major. Cell Microbiol 13: 274–283. 20964798 10.1111/j.1462-5822.2010.01534.x

[12] Rougeron V, De Meeus T, Kako Ouraga S, Hide M, Banuls AL (2010) “Everything you always wanted to know about sex (but were afraid to ask)” in Leishmania after two decades of laboratory and field analyses. PLoS Pathog 6: e1001004. 20808896 10.1371/journal.ppat.1001004 PMC2924324

[13] Akopyants NS, Kimblin N, Secundino N, Patrick R, Peters N, et al (2009) Demonstration of genetic exchange during cyclical development of Leishmania in the sand fly vector. Science 324: 265–268. 19359589 10.1126/science.1169464 PMC2729066

[14] Jenni L, Marti S, Schweizer J, Betschart B, Le Page RW, et al (1986) Hybrid formation between African trypanosomes during cyclical transmission. Nature 322: 173–175. 3724860 10.1038/322173a0

[15] Sadlova J, Yeo M, Seblova V, Lewis MD, Mauricio I, et al (2011) Visualisation of Leishmania donovani fluorescent hybrids during early stage development in the sand fly vector. PLoS One 6: e19851. 21637755 10.1371/journal.pone.0019851 PMC3103508

[16] Capul AA, Barron T, Dobson DE, Turco SJ, Beverley SM (2007) Two functionally divergent UDP-Gal nucleotide sugar transporters participate in phosphoglycan synthesis in Leishmania major. J Biol Chem 282: 14006–14017. 17347153 10.1074/jbc.M610869200 PMC2807729

[17] Sacks D, Kamhawi S (2001) Molecular aspects of parasite-vector and vector-host interactions in leishmaniasis. Annu Rev Microbiol 55: 453–483. 11544364 10.1146/annurev.micro.55.1.453

[18] Lukes J, Mauricio IL, Schonian G, Dujardin JC, Soteriadou K, et al (2007) Evolutionary and geographical history of the Leishmania donovani complex with a revision of current taxonomy. Proc Natl Acad Sci U S A 104: 9375–9380. 17517634 10.1073/pnas.0703678104 PMC1890502

[19] Walters LL, Irons KP, Chaplin G, Tesh RB (1993) Life cycle of Leishmania major (Kinetoplastida: Trypanosomatidae) in the neotropical sand fly Lutzomyia longipalpis (Diptera: Psychodidae). J Med Entomol 30: 699–718. 8360894 10.1093/jmedent/30.4.699

[20] Aslan H, Dey R, Meneses C, Castrovinci P, Bezerra Jeronimo SM, et al (2013) A New Model of Progressive Visceral Leishmaniasis in Hamsters by Natural Transmission via Bites of Vector Sand Flies. J Infect Dis 207 8: 1328–38. 23288926 10.1093/infdis/jis932 PMC3603531

[21] Cruz AK, Titus R, Beverley SM (1993) Plasticity in chromosome number and testing of essential genes in Leishmania by targeting. Proc Natl Acad Sci U S A 90: 1599–1603. 8381972 10.1073/pnas.90.4.1599 PMC45922

[22] Turner CM, Hide G, Buchanan N, Tait A (1995) Trypanosoma brucei: inheritance of kinetoplast DNA maxicircles in a genetic cross and their segregation during vegetative growth. Exp Parasitol 80: 234–241. 7895834 10.1006/expr.1995.1029

[23] Heitman J (2010) Evolution of eukaryotic microbial pathogens via covert sexual reproduction. Cell Host Microbe 8: 86–99. 20638645 10.1016/j.chom.2010.06.011 PMC2916653

[24] Gaunt MW, Yeo M, Frame IA, Stothard JR, Carrasco HJ, et al (2003) Mechanism of genetic exchange in American trypanosomes. Nature 421: 936–939. 12606999 10.1038/nature01438

[25] Gibson W (2001) Sex and evolution in trypanosomes. Int J Parasitol 31: 643–647. 11334957 10.1016/s0020-7519(01)00138-2

[26] Peacock L, Ferris V, Sharma R, Sunter J, Bailey M, et al (2011) Identification of the meiotic life cycle stage of Trypanosoma brucei in the tsetse fly. Proc Natl Acad Sci U S A 108: 3671–3676. 21321215 10.1073/pnas.1019423108 PMC3048101

[27] Ivens AC, Peacock CS, Worthey EA, Murphy L, Aggarwal G, et al (2005) The genome of the kinetoplastid parasite, Leishmania major. Science 309: 436–442. 16020728 10.1126/science.1112680 PMC1470643

[28] Gibson W, Bailey M (1994) Genetic exchange in Trypanosoma brucei: evidence for meiosis from analysis of a cross between drug-resistant transformants. Mol Biochem Parasitol 64: 241–252. 7935602 10.1016/0166-6851(94)00017-4

[29] Tait A, Macleod A, Tweedie A, Masiga D, Turner CM (2007) Genetic exchange in Trypanosoma brucei: evidence for mating prior to metacyclic stage development. Mol Biochem Parasitol 151: 133–136. 17134768 10.1016/j.molbiopara.2006.10.009 PMC2311417

[30] Pan J, Snell WJ (2000) Signal transduction during fertilization in the unicellular green alga, Chlamydomonas. Curr Opin Microbiol 3: 596–602. 11121779 10.1016/s1369-5274(00)00146-6

[31] Martinez-Calvillo S, Sunkin SM, Yan S, Fox M, Stuart K, et al (2001) Genomic organization and functional characterization of the Leishmania major Friedlin ribosomal RNA gene locus. Mol Biochem Parasitol 116: 147–157. 11522348 10.1016/s0166-6851(01)00310-3

[32] Marchand M, Daoud S, Titus RG, Louis J, Boon T (1987) Variants with reduced virulence derived from Leishmania major after mutagen treatment. Parasite Immunol 9: 81–92. 3562060 10.1111/j.1365-3024.1987.tb00490.x

[33] Neva FA, Wyler D, Nash T (1979) Cutaneous leishmaniasis–a case with persistent organisms after treatment in presence of normal immune response. Am J Trop Med Hyg 28: 467–471. 222157

[34] Stamper LW, Patrick RL, Fay MP, Lawyer PG, Elnaiem DE, et al (2011) Infection parameters in the sand fly vector that predict transmission of Leishmania major. PLoS Negl Trop Dis 5: e1288. 21886852 10.1371/journal.pntd.0001288 PMC3160291

[35] Saraiva EM, Pimenta PF, Brodin TN, Rowton E, Modi GB, et al (1995) Changes in lipophosphoglycan and gene expression associated with the development of Leishmania major in Phlebotomus papatasi. Parasitology 111: 275–287. 7567096 10.1017/s003118200008183x

[36] Li H, Handsaker B, Wysoker A, Fennell T, Ruan J, et al (2009) The Sequence Alignment/Map format and SAMtools. Bioinformatics 25: 2078–2079. 19505943 10.1093/bioinformatics/btp352 PMC2723002

[37] Hothorn T, Hornik K, van de Wiel MAV, Zeileis A (2008) Implementing a Class of Permutation Tests: The coin Package. Journal of Statistical Software 28: 1–23.

