# Supplementary figures and images for: The Mating Competence of Geographically Diverse Leishmania major Strains in Their Natural and Unnatural Sand Fly Vectors
Source: PLoS Genet. 2013 Jul 25;9(7):e1003672. doi: 10.1371/journal.pgen.1003672 (PMC3723561; doi:10.1371/journal.pgen.1003672)

Fig S1

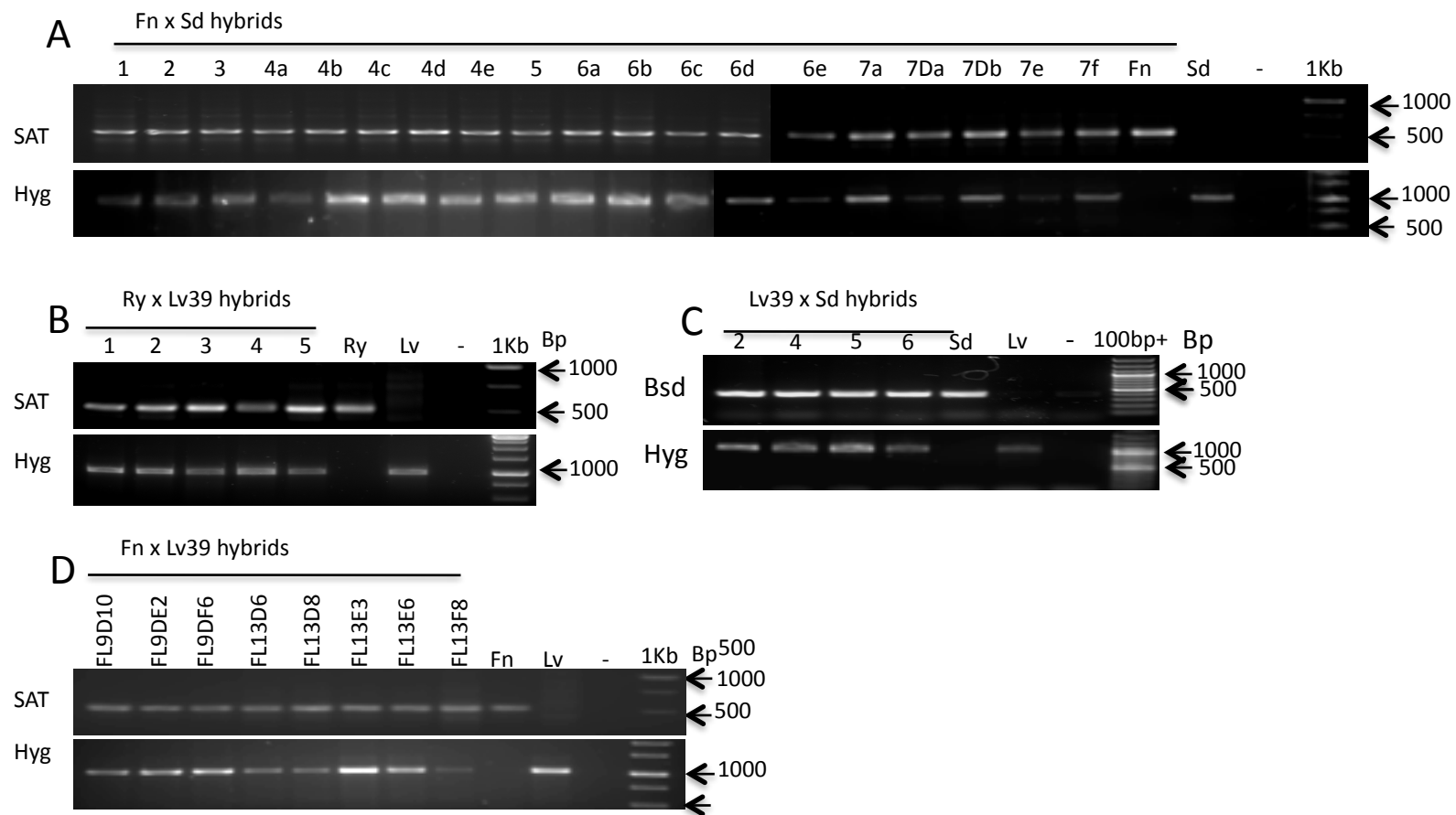

Supplement: Figure S1 — PCR for parental selectable drug markers. Samples are (A) Fn, Fn/Sat; Sd, Sd/Hyg; -, no template control; (B) Ry, Ry/Sat; Lv, Lv39/Hyg; -, no template control; (C) Lv, Lv39/Hyg; Sd, Sd/BSD; -, no template control; (D) Fn, Fn/Sat; Lv, Lv39/Hyg; -, no template control. (PDF) [file pgen.1003672.s001.pdf]

Fig S2

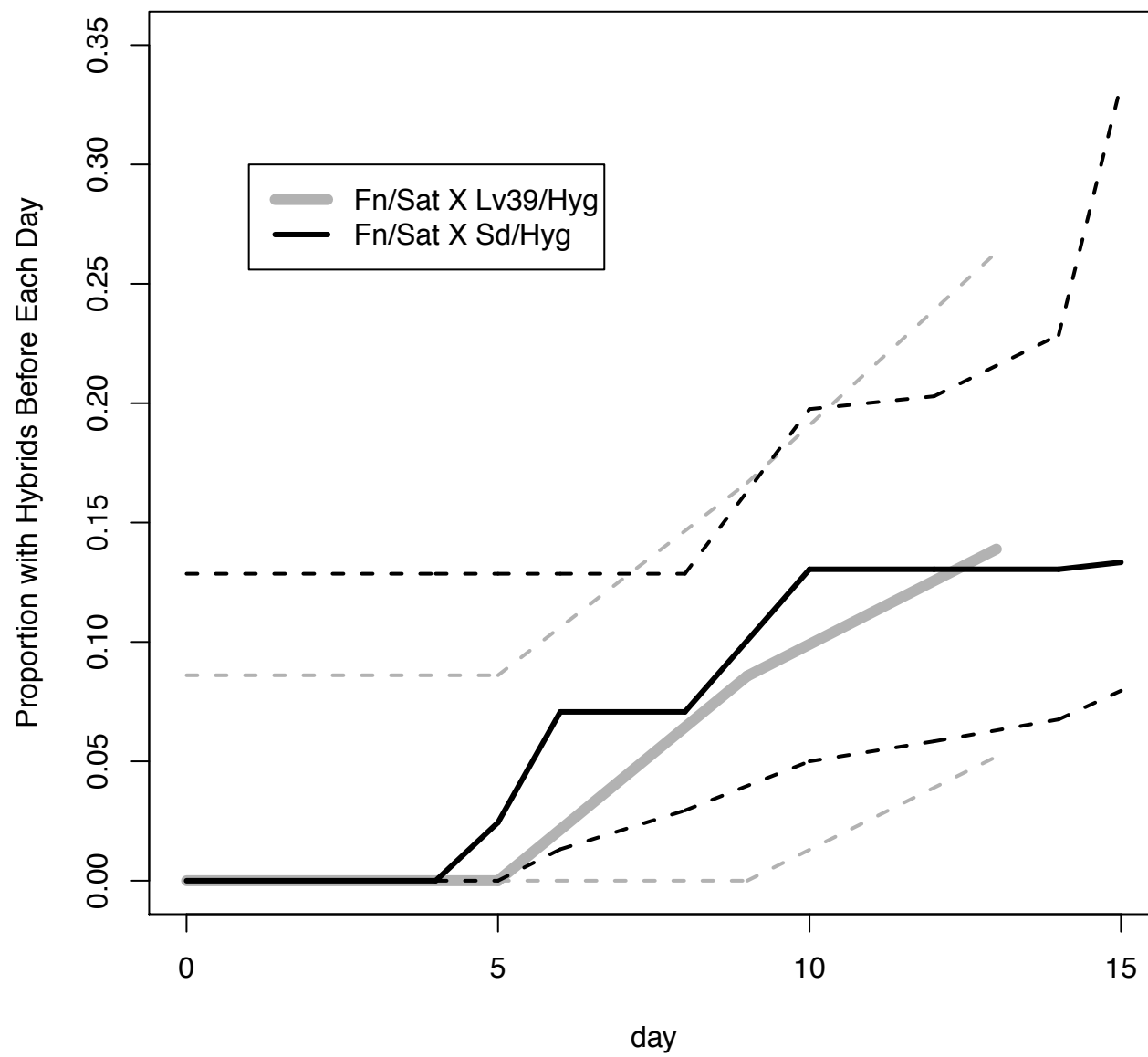

Supplement: Figure S2 — Plot of nonparametric maximum likelihood estimators of the proportion of flies with any hybrid by day for each of the crosses (solid lines), along with 95% pointwise confidence intervals (dotted lines). The confidence intervals use nonparametric bootstrap with a necessary adjustment for the upper interval for early days using an exact binomial upper interval. (PDF) [file pgen.1003672.s002.pdf]

Fig S3

Fn/Sat X Sd/Hyg  
LmjF.34.0080

Fn/Sat X Lv39/Hyg  
LmjF.31.0020

Sd/BsdX Lv39/Hyg  
LmjF.35.0050

Ry/Sat X Lv39/Hyg  
LmjF.21.0040

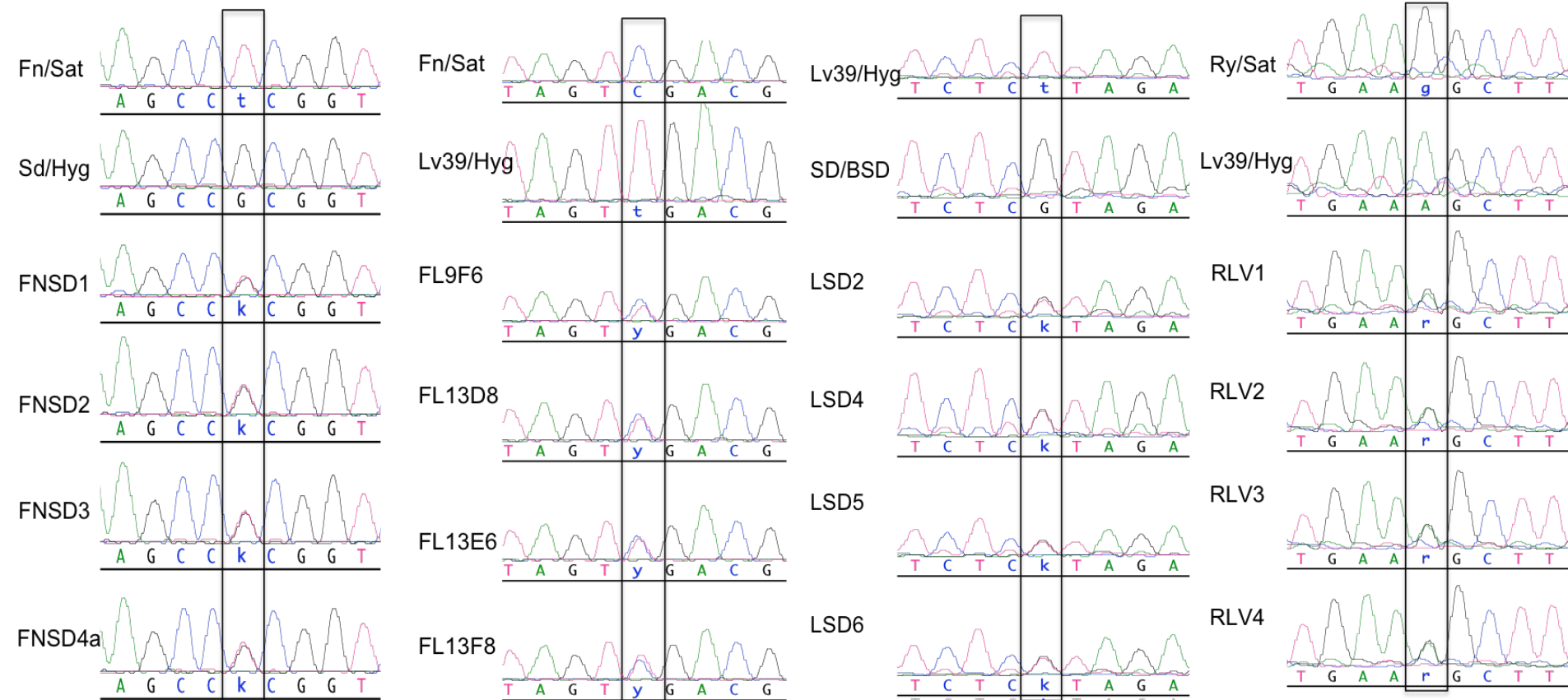

Supplement: Figure S3 — DNA sequence traces of PCR-amplified chromosomal genes showing inheritance of both parental alleles. DNA sequence traces are shown for 4 representative loci and 16 representative 2n hybrids and their respective parents. SNPs are identified by the boxed regions. (PDF) [file pgen.1003672.s003.pdf]

Fig S4

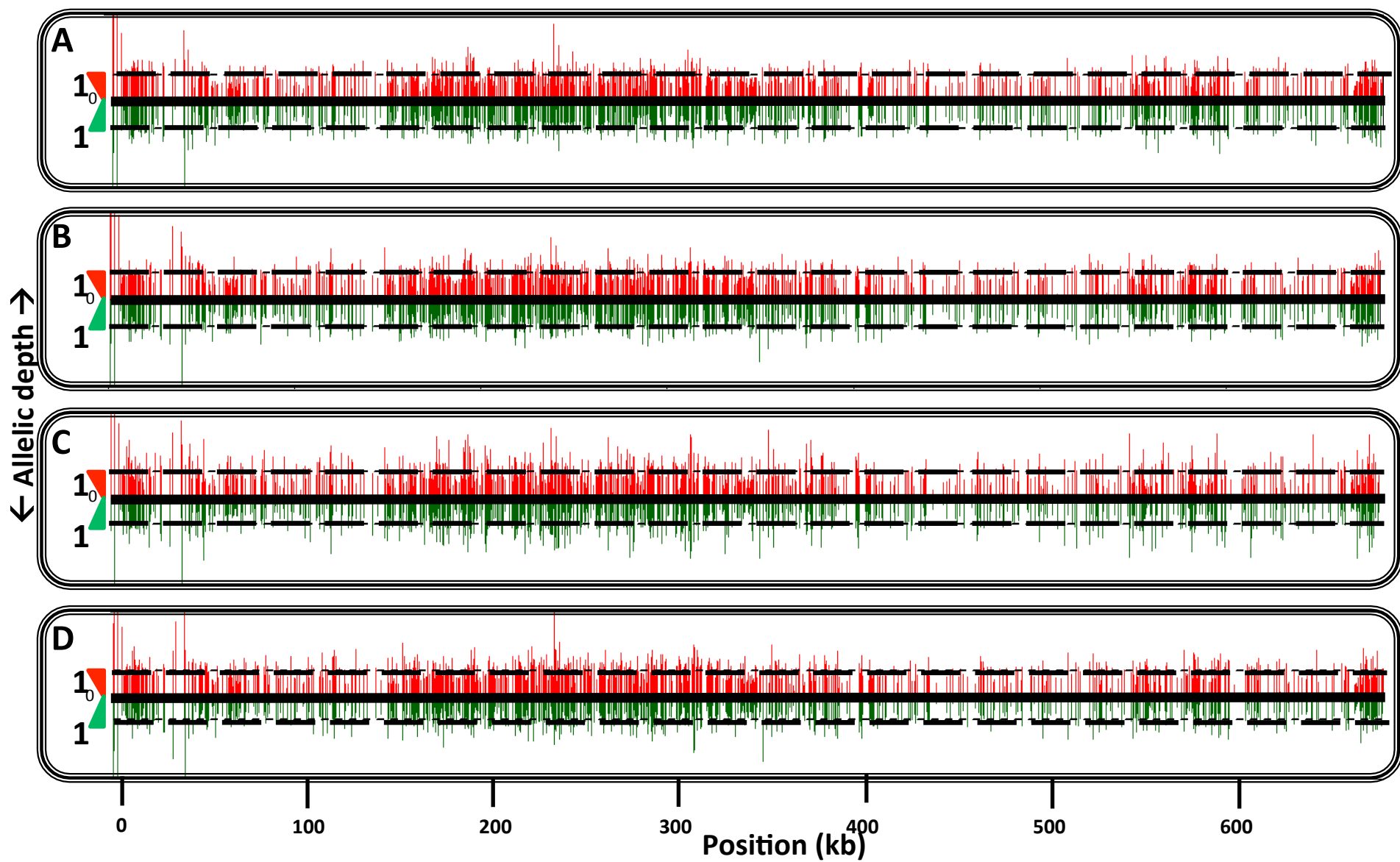

Supplement: Figure S4 — Comparison of homozygous parental SNPs on chromosome 17 for four additional representative hybrids. Please refer to the legend for Figure 3 for a description of the methods and symbols used. Panel A and B, hybrids recovered from P. duboscqi (1_10_B12 and 4_7_A3). Panel C and D, hybrids recovered from L. longipalpais (LL1_3 and 3L3c1). (PDF) [file pgen.1003672.s004.pdf]

Fig S5

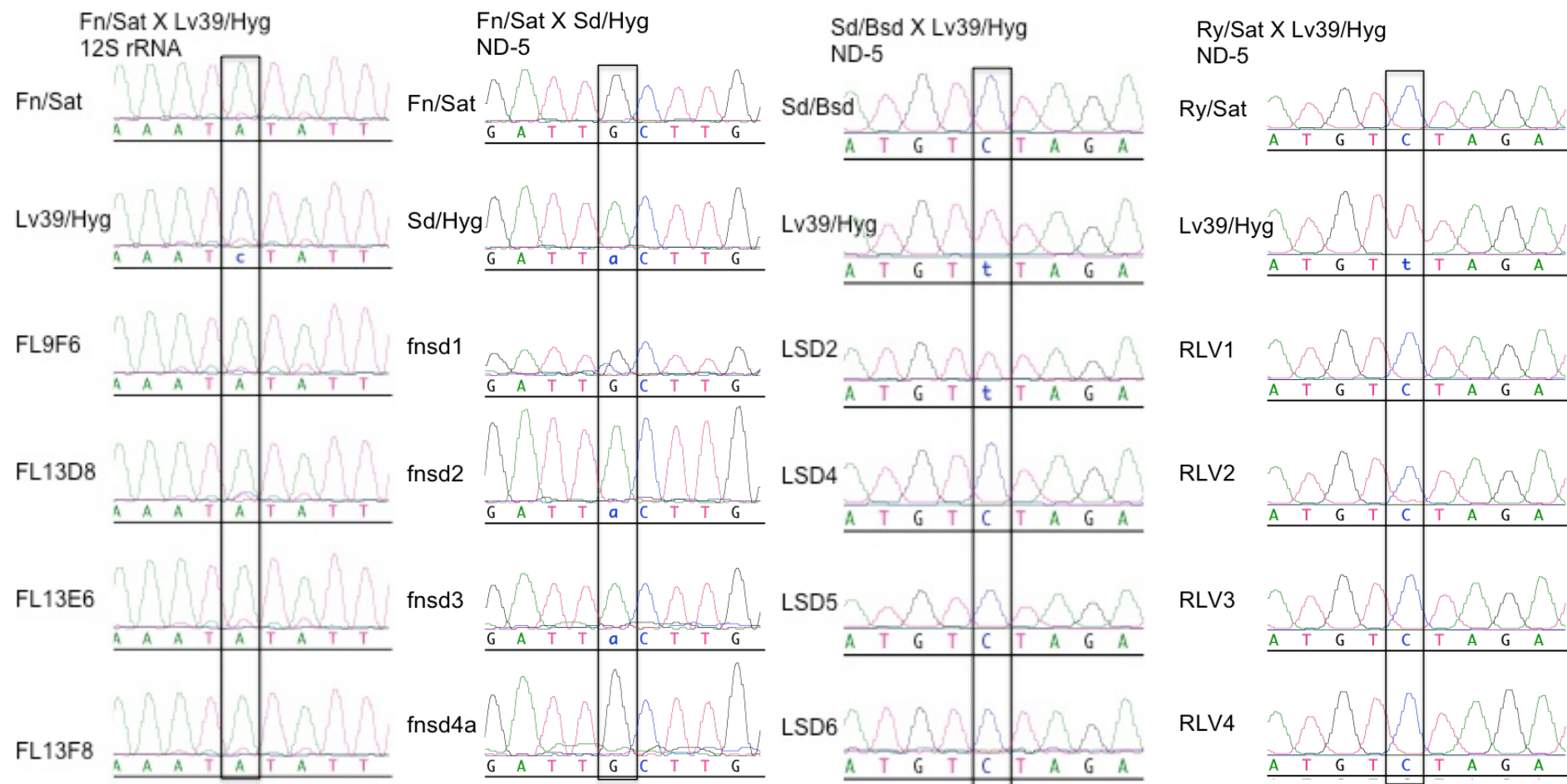

Supplement: Figure S5 — DNA sequence traces of PCR-amplified maxicircle genes showing inheritance of a single parental allele. DNA sequence traces are shown for representative loci in the maxicircle genes (1 SNP in the 12S rRNA locus, and 2 SNPs in the ND-5 locus) and 16 representative 2n hybrids and their respective parents. SNPs are shown in boxed regions. (PDF) [file pgen.1003672.s005.pdf]

Fig S6

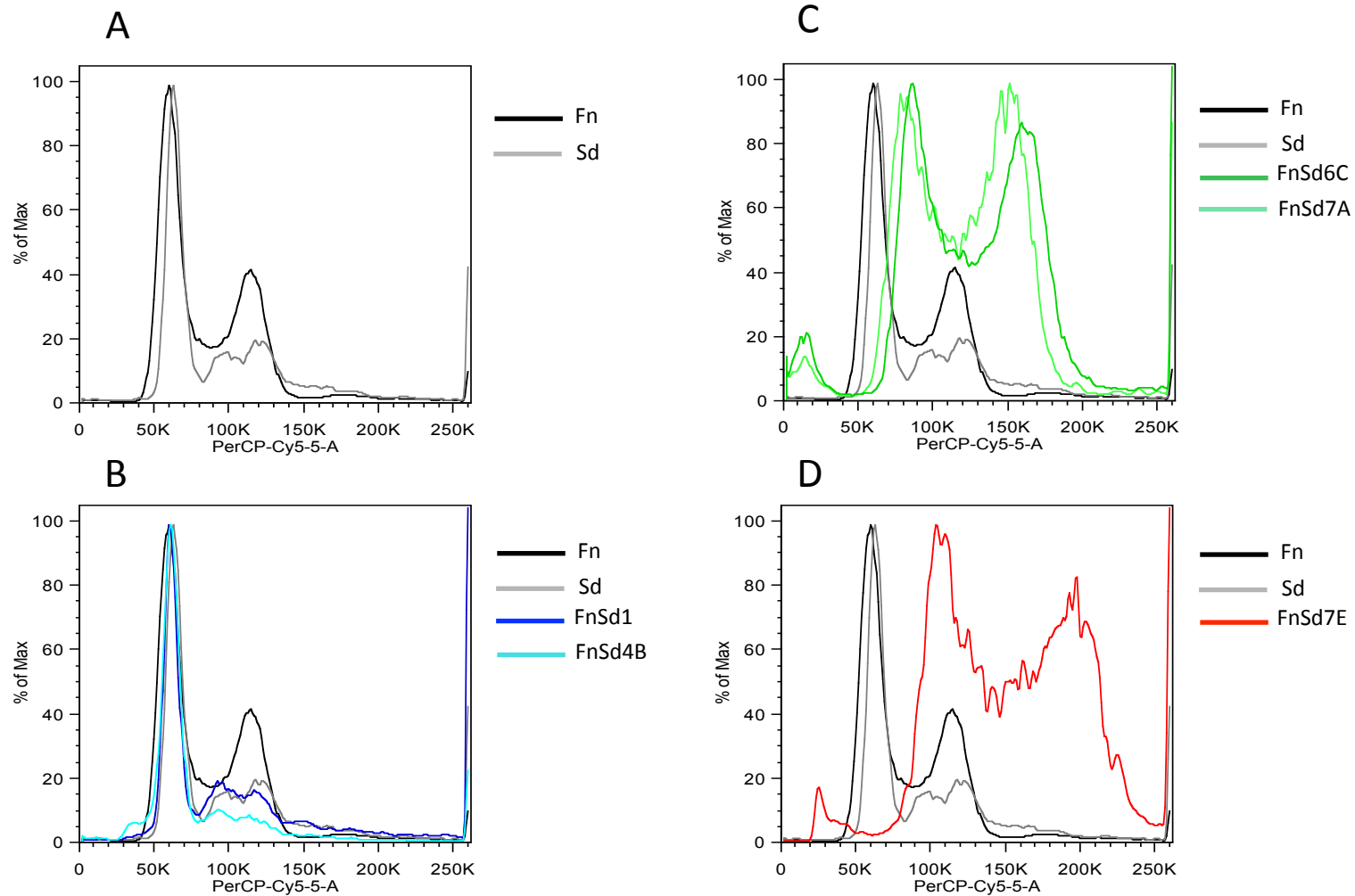

Supplement: Figure S6 — DNA contents of parental Fn/Sat and Sd/Hyg (A) , and representative ‘2n’ (B), ‘3n’ (C), and ‘4n’ (D) hybrid clones. DNA content was measured in log phase cells by flow cytometry after staining RNase treated permeabilized cells with propidium iodide. (PDF) [file pgen.1003672.s006.pdf]

Fig S7

A

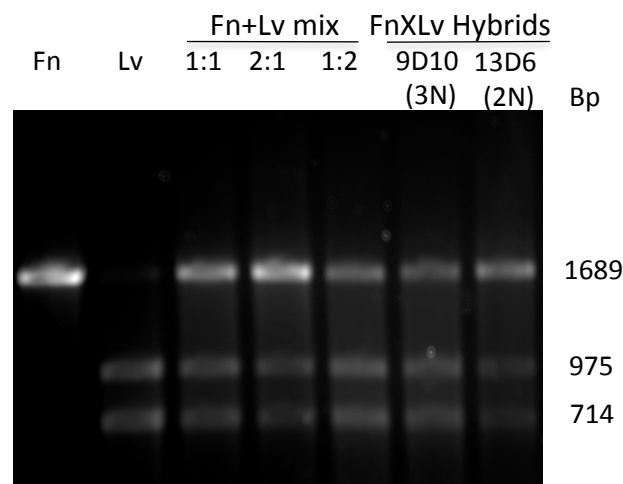

B

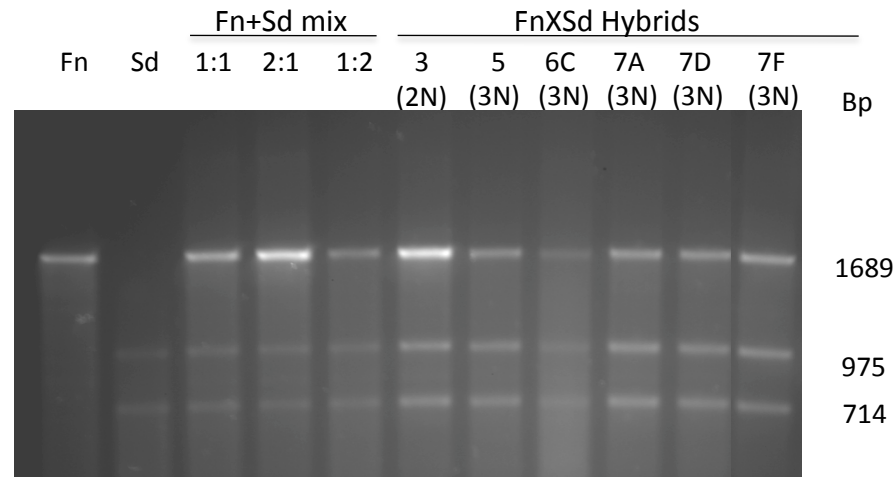

C

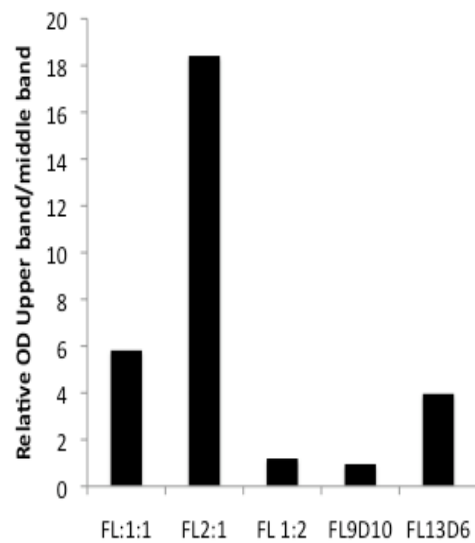

D

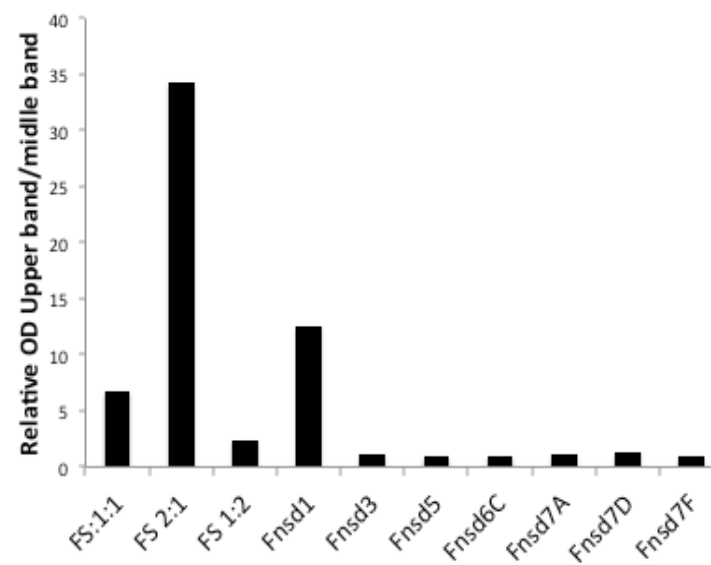

Supplement: Figure S7 — SNP-CAPS analysis of triploid hybrids. (A,B) Digestion with SACII of the G6PD-LmjF34.0080 divergent region PCR product is shown. (C,D) The graph shows the ratio between the intensity of the uncut upper band from Fn (F) and the middle band from either Lv39 (L) or Sd (S), and compares the various hybrids with controls. (PDF) [file pgen.1003672.s007.pdf]
