# Supplementary material for: The Mating Competence of Geographically Diverse Leishmania major Strains in Their Natural and Unnatural Sand Fly Vectors
Source: PLoS Genet. 2013 Jul 25;9(7):e1003672. doi: 10.1371/journal.pgen.1003672 (PMC3723561; doi:10.1371/journal.pgen.1003672)
Supplement: Table S1 — The PCR primer sequences for each allelic marker, their chromosomal location, and the conditions used for their amplification are shown. Δ - Genebank: M10126.1; nd, not determined. (PDF) [file pgen.1003672.s008.pdf]

Table S1. PCR primers and conditions

Gene ID	Chr	Chromosomal location	Fwd primer	Rev primer	Fragment length (bp)	Annealing Temp(^{0}C)	Ref
Hygromycin B (Hyg)	24	nd	ATGAAAAGCCTG AACTCACC	CTATTCCTTTGCC CTCGG	1029	62	This work
Nourseothricin (Sat)	27	nd	ATGAAGATTTCG GTGATCCC	TTAGGCGTCATC CTGTGC	525	68	This work
Blasticidin (Bsd)	18	nd	ATGCCTTTGTCTC AAGAAGAATC	TTAGCCCTCCCA CACATAAC	393	68	This work
LmjF.02.0085	2	28965-29909	ATGCCGCGCCGA TACAGACACAT	CTGCTTCAGCCA TGATGCACGC	922	71.5	[13]
LmjF04.0070	4	29378-30900	CGRCTAGAAACG CAGAGTAC	CACGTGATCTGC ACAAACAC	599	60	This work
LmjF09.0740	9	288100-289100	TACAGGGTGGAG AGRTCYAT	GCATGCTGCAGT ACCTKTTG	637	60	This work
LmjF10.0290*	10	130280-131280	TACTTYGACCTCA GCGTGAC	GATGGTGTTCTT CGTCGTCA	518	60	This work and [18]
LmjF14.0130	14	32470-33230	GAATGGAGAACM TSGGCAAG	GAAGCGATCCAG AATGCTST	739	60	This work and [18]
LmjF21.0040	21	16381-17118	TGGCGCCGTCTA CCTGCGAC	GAATGCGTTGAG AGCGTCTG	578	55	[13]
LinJ.25.2530	25	872123 - 872908	ATGGCGGAGCTC CTTAGCAAAAAG	CCATCTTTGCTG GGTCCTTGC	778	68	[13]
LmjF31.0020	31	7700-8700	AAGAACATGCCRA RAAGGGC	CCGAGTTCTTCG GMWCGTTT	700	60	This work
LmjF.31.3150	31	1465014-1469423	GAAAGCTGATGAA GCTGCTGGA	CTAGCGCGTCTG CTTCGACACAAC	530	55	[13]
LmjF.31.3110	31	1454006-1456546	GCTCTGTGCGGAA GCGTATCCC	CTAGCAGCACGC GCTCTTCACCT	749	71.5	[13]
LmjF.34.0080	34	26530-27530	GATYCGMGAGAA GGAGAATG	CGGTCGTTGTTG ATGTTGAG	684	60	This work and [18]
LmjF.35.0050	35	17610-18710	ATGCGTCGCCTCT TTGATGCCTC	AAGTGCACCACG GACTTGATAGC	1100	71.5	[13]
LmjF.35.3340	35	1363367-1364500	TGTGAGCHTGGCR AGAATCT	GTATCACAACGC TGGGGAGT	697	60	This work and [18]

LmjF.36.0050	36	17464-19125	ATGTCGTCAGAGG AGAAGTTAACAGC	GATTGGTGTAGT TCGGTGCGTG	1018	71.5	[13]
CYTB[Δ]	Maxi circle	5403-6481	AGCGGAGAGRAR AGAAAAGG	GYTCRCAATAAA ATGCAAATC	618	60	This work
12S rRNA[Δ]	Maxi circle	438-1610	AACTARTGAWGG CACAGTTGTTCT	ACCCAACTAACG AATTGCWTTT	818	59	This work
Nd5[Δ]	Maxi circle	15626-15645	GAYGCDATGGAAG GACCDAT	CCACAYAAAAAY CAYAANGAACA	456	59	This work

Δ - Genebank: M10126.1; nd, not determined
