# Supplementary material for: The Mating Competence of Geographically Diverse Leishmania major Strains in Their Natural and Unnatural Sand Fly Vectors
Source: PLoS Genet. 2013 Jul 25;9(7):e1003672. doi: 10.1371/journal.pgen.1003672 (PMC3723561; doi:10.1371/journal.pgen.1003672)
Supplement: Table S2 — The chromosomal location, position and description of each SNP are shown. Position of SNPs in the maxicircle genes refer to L .tarentolae kDNA. Genbank: M10126.1. (PDF) [file pgen.1003672.s009.pdf]

Table S2. SNP positions and descriptions

Chromosome	Locus/ chromosome	Number of analyzed SNPs/ locus	SNP Position/ description
**Fn/Sat x Sd/Hyg**			
14	LmjF14.0130	1	370/ F=G S=A
34	LmjF.34.0080	2	777/ F=C S=T<br>975/ F=T S=G
35	LmjF35.3340	1	660/F=C S=R*
36	LmjF36.0050	1	390/ F=C S=T
Maxicircle	12SrRNA	1	458/ F=A S=C
	ND-5	1	831/ F=G S=A
**Fn/Sat x Lv39c5/Hyg**			
4	LmjF.04.0070	1	465/ F=T L=C
10	LmjF.10.0290	1	657/ F=C L=T
31	LmjF.31.0020	1	700 F=C L=T
34	LmjF.34.0080	2	777/ F=C L=T<br>975/ F=T L=G
35	LmjF.35.0050	1	816/ F=T L=C
Maxicircle	12SrRNA	1	456/ F=A L=C
	CYTB	1	106/ F=G L=A
**Lv39c5/Hyg x Sd/BSD**			
2	LmjF.02.0085	2	739/ S=A L=G<br>608/ S=G L=C
9	LmjF09.0740	1	6360/ S=C L=T
21	LmjF.21.0040	1	345/ S=G L=A
31	LmjF.31.3110	1	2103/ S=G L=A
35	LmjF.35.0050	1	727/ S=G L=T
36	LmjF.36.0050	1	282/ S-T L=C
Maxicircle	ND-5	1	831/ S=A L=G<br>958/ S=C L=T
**Ry/Sat x Lv39c5/Hyg**			
21	LmjF21.0040	1	345/ R=G L=A
25	LmjF.25.2420	1	264/ R=A L=G
31	LmjF.31.3150	1	4122/ R=G L=T
35	LmjF35.0050	1	727/ R=G L=T
36	LmjF36.0050	1	632/ R=A L=G
Maxicircle	ND-5	2	909/ R=C L=T<br>1080/ R=C L=T
	CYTB	2	352/ R=C L=T<br>495/ R=T L=C

Position of SNPs in the maxicircle genes refer to *L .tarentolae* kDNA. Genbank: M10126.1
